# Ontology of the apelinergic system in mouse pancreas during pregnancy and relationship with β-cell mass

**DOI:** 10.1038/s41598-021-94725-0

**Published:** 2021-07-29

**Authors:** Brenda Strutt, Sandra Szlapinski, Thineesha Gnaneswaran, Sarah Donegan, Jessica Hill, Jamie Bennett, David J. Hill

**Affiliations:** 1 Lawson Health Research Institute, St Joseph Health Care, 268 Grosvenor St, London, ON N6A 4V2 Canada; 2 Department of Physiology and Pharmacology, Western University, London, ON N6A 3K7 Canada; 3 Institute of Biomedical and Clinical Science, University of Exeter Medical School, Exeter, EX2 5DW UK; 4 Life Sciences Program, School of Interdisciplinary Science, McMaster University, Hamilton, ON L8S 4LD Canada; 5 Departments of Medicine and Paediatrics, Western University, London, ON N6A 3K7 Canada

**Keywords:** Gestational diabetes, DNA synthesis, Cell growth

## Abstract

The apelin receptor (Aplnr) and its ligands, Apelin and Apela, contribute to metabolic control. The insulin resistance associated with pregnancy is accommodated by an expansion of pancreatic β-cell mass (BCM) and increased insulin secretion, involving the proliferation of insulin-expressing, glucose transporter 2-low (Ins^+^Glut2^LO^) progenitor cells. We examined changes in the apelinergic system during normal mouse pregnancy and in pregnancies complicated by glucose intolerance with reduced BCM. Expression of Aplnr, Apelin and Apela was quantified in Ins^+^Glut2^LO^ cells isolated from mouse pancreata and found to be significantly higher than in mature β-cells by DNA microarray and qPCR. Apelin was localized to most β-cells by immunohistochemistry although Aplnr was predominantly associated with Ins^+^Glut2^LO^ cells. Aplnr-staining cells increased three- to four-fold during pregnancy being maximal at gestational days (GD) 9–12 but were significantly reduced in glucose intolerant mice. Apelin-13 increased β-cell proliferation in isolated mouse islets and INS1E cells, but not glucose-stimulated insulin secretion. Glucose intolerant pregnant mice had significantly elevated serum Apelin levels at GD 9 associated with an increased presence of placental IL-6. Placental expression of the apelinergic axis remained unaltered, however. Results show that the apelinergic system is highly expressed in pancreatic β-cell progenitors and may contribute to β-cell proliferation in pregnancy.

## Introduction

The physiology of pregnancy tests the metabolic plasticity of the mother and initiates adaptive responses to metabolic stress. Within the human pancreas, substantial increases in β-cell mass (BCM) normally occur in second and third trimester preceding the appearance of insulin resistance^1,2^. A failure of β-cells to adaptively expand after the first trimester may place the mother at risk of developing GDM^3^ associated with elevated levels of pro-inflammatory cytokines^4–6^ which contribute to β-cell dysfunction^7^. Similarly, β-cell mitogenesis is normally low in adult mice but increases during pregnancy contributing to a two- to three-fold increase in BCM^8^. In rodents this has been linked to the mitogenic effects of prolactin and placental lactogen (PL) on β-cells^8–11^, both of which increase across pregnancy in the maternal circulation^9^. Targeted over-expression of PL in mouse β-cells resulted in their increased proliferation^11^, mediated by prolactin receptors. Conversely, targeted deletion of the prolactin receptor prevented a gestational increase in BCM, impaired insulin release and led to glucose intolerance^12,13^.

An increase in β-cells during pregnancy occurs partly through self-renewal of existing, mature β-cells. In rodents the lifespan of the β-cell in adult life is around 58 days^14^. An increased rate of proliferation during pregnancy without a change in apoptotic rate results in an accumulation of additional β-cells. However, new β-cells may also derive from a number of progenitor phenotypes during pregnancy. These include insulin-expressing cells that do not express the Fltp gene, a marker of functional β-cells^15^, which are highly proliferative and which may also express the platelet-derived growth factor (PDGF) receptor-α^16^. A separate type of multi-lineage progenitor has been identified in mouse and human pancreata throughout life, both within islets and in the small, extra-islet endocrine clusters^17^. This progenitor cell fraction expresses some insulin, but glucose-stimulated insulin secretion (GSIS) is poor due to low expression of glucose transporter 2 (Ins^+^Glut2^LO^ cells)^18^, although they have the capacity to differentiate into functional β-cells in vitro^19^. Such cells relatively lack β-cell maturity markers such as expression of the transcription factors MafA and Nkx6.1, while over-expressing progenitor cells markers such as neurogenin-3 and MafB^18,19^. During mouse pregnancy the percentage of Ins^+^Glut2^LO^ cells that are proliferating increases significantly at gestational day (GD) 9 preceding the increase in BCM^20^. However, in a mouse model of gestational glucose intolerance characterized by a sub-optimal increase in BCM the number of proliferating Ins^+^Glut2^LO^ cells was significantly lower^21^. Neogenesis of new β-cells is also likely during human pregnancy due to the reappearance of C-peptide in women with long-standing type 1 diabetes where residual surviving β-cells are expected to be scarce^22^.

The local trophic factors contributing to the proliferation and differentiation of β-cell progenitors during pregnancy are not well characterized but may include locally expressed paracrine molecules such as Apelin (Apln) and Apela [Elabela]. Apelin and Apela are endogenous ligands for the G-protein coupled receptors, Aplnr (APJ)^23,24^ and GPR25^25^, and both Apelin and the Aplnr are found in multiple tissue types, including pancreas^26^. The apelinergic system is active in the fetoplacental unit and is thought to promote transplacental glucose transport^27^. Additionally, Apela is morphogenic for embryonic cardiovascular system formation and early placental development, while Apelin acts in mid or late gestation to mediate fetal angiogenesis and energy homeostasis^28^. Apelin is released by the placental syncytiotrophoblast into the maternal circulation with concentrations increasing throughout pregnancy in both humans and rodents^29,30^.

The apelinergic axis may also modulate metabolism since adipose-derived Apelin has been associated with increased glucose uptake and insulin sensitivity^28,31,32^. Furthermore, Apelin gene-null mice demonstrate a decreased insulin sensitivity and hyperinsulinemia, which could be reversed by Apelin administration, as was similarly reported in the db/db mouse model of type 2 diabetes^32^. Interestingly, patients who are obese or have type 2 diabetes show increased circulating Apelin levels, which suggests the possibility of Apelin resistance^33,34^. Similarly, obese and insulin-resistant pregnant rats had increased circulating and placental Apelin levels at term^35^. However, altered Apelin levels were not associated with a clinical diagnosis of gestational diabetes^36^.

Within the pancreas, apelin has been localized to, and is released from, β-cells^37^ and may influence ß-cell number since targeted deletion of the Aplnr from mouse β-cells resulted in a reduced BCM and impaired glucose-stimulated insulin secretion (GSIS)^38^. Conversely, treatment with apelin protected against cellular stress and promoted β-cell survival in the Akita mouse model of type 1 diabetes^39^. Additionally, a long-acting depot of apelin reversed insulin resistance and promoted β-cell proliferation in diabetic rats^40^. Apelin expression has also been associated in other tissues with progenitor cells^41^, which suggests that it might be involved in the expansion and/or differentiation of Ins^+^Glut2^LO^ cells. Taken together, these findings suggest that the apelinergic axis could contribute to the increase in BCM during pregnancy, which we have examined in the present studies.

## Results

### Differential gene expression in pancreatic Ins^+^Glut2^LO^ cells

We analyzed differential gene expression in Ins^+^Glut2^LO^ vs. Ins^+^Glut2^HI^ cells by DNA microarray following separation by FACS from pancreata of 7-day old neonatal mice. A total of 262 genes were identified where the relative levels of expression were higher by at least tenfold in Ins^+^Glut2^LO^ cells (Supplementary Table 1). Partek GO enrichment software revealed gene clusters known to be associated with cell lineage commitment, cell proliferation, angiogenesis, and extracellular matrix modeling (Table 1). Within the genes with the greatest differential expression in Ins^+^Glut2^LO^ cells were Apelin and Aplnr.

**Table 1:** Fold increase in selected genes over-expressed at least ten-fold in Ins^+^Glut2^LO^ cells relative to Ins^+^Glut2^HI^ cells isolated from pancreata of 7-day old mice. Expressed genes are grouped relative to biological roles reported previously.

**β-cell development**		**Cell lineage commitment**	
Aplnr (APJ)	48	Efnb2	20
Apln (Apelin)	14	Sox4	11
Fabp4	24	Sox18	39
Dlk1	10	Fzd4	18
**Cell proliferation**		**Extracellular matrix**	
Igfbp5	15	Eln	33
Igfbp3	13	Lamb1	21
Pdgfra	12	Thbs1	15
Pdgfrb	14	Hspg2	26
		Dcn	16
**Angiogenesis**		Mmp2	14
Plvap	67	Mmp14	12
Ednra	12	Col1a2	18
Ece1	11	Col14a1	11
Esam	10	Col14a2	29
Flt1	21	Col2a1	19
		Col4a1	25
		Col61a	11

A full analysis of differential gene expression is shown in Supplementary Table 1.

*Efnb2* Ephrin-B2, *Fzd4* frizzled-4, *Igfbp* IGF binding proteins 3, *Pdgfr* platelet-derived growth factor receptors, *Plvap* plasmalemma vesicle associated protein, *Ednra* endothelin receptor type A, *Ece1* endothelin converting enzyme 1, *Esam* endothelial cell adhesion molecule, *Flt-1* Fms related tyrosine kinase 1, *Eln* tropoelastin, *Lamb1* liver fibrosis-specific gene, *Thbs1* thrombospondin 1, *Hspg2* heparan sulfate proteoglycan 2, *Dcn* decorin, *Mmp* matrix metallopeptidases, *Col* collagen genes, *Dlk1* delta like non-canonical notch ligand 1, *Fabp4* fatty acid binding protein-4, *Apln* Apelin, *Aplnr* apelin receptor.

**Figure 1:**
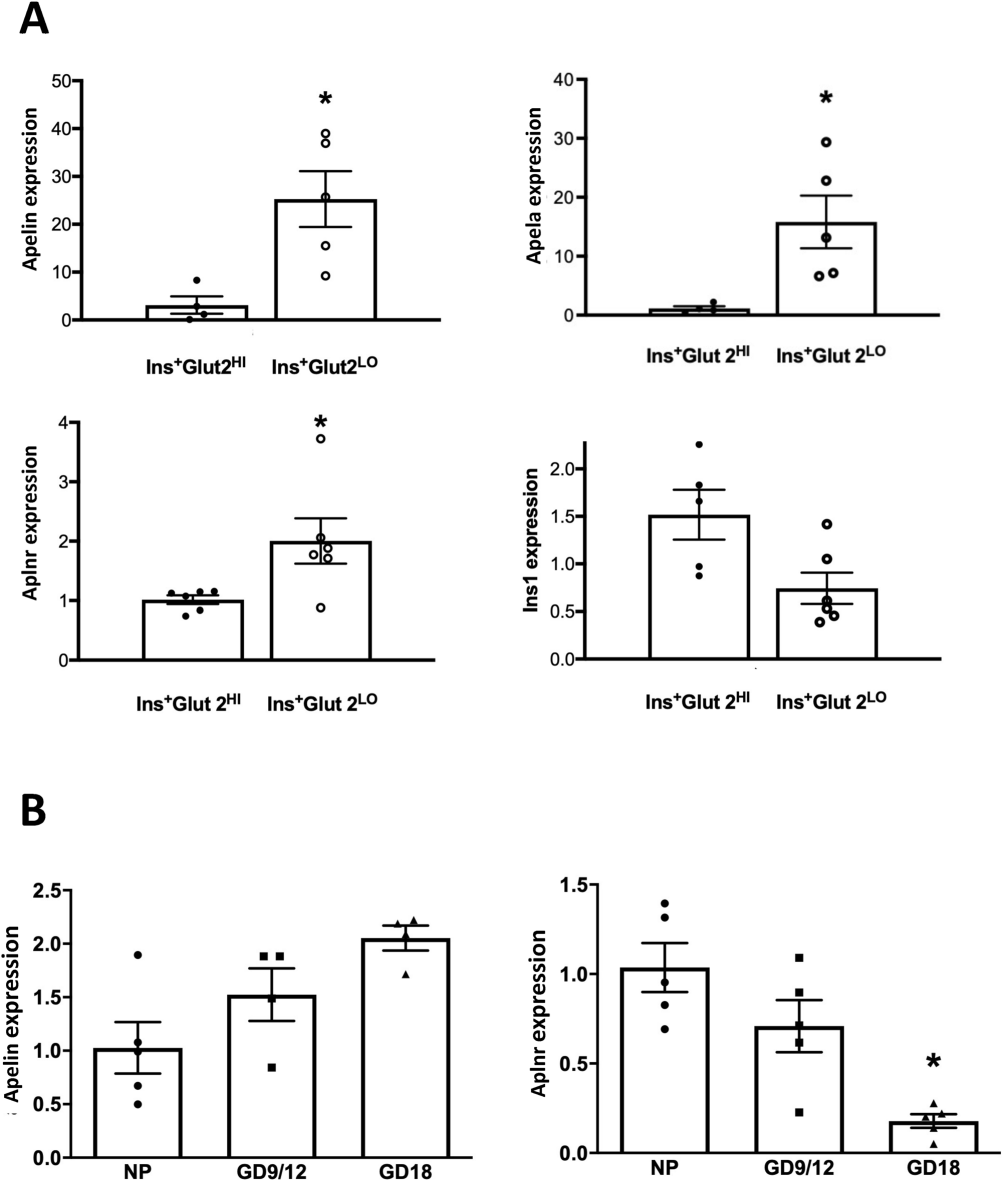
(**A**) Relative expression levels of mRNA for Apelin, Apela, Aplnr and insulin (INS1) quantified by qPCR in Ins^+^Glut2^HI^ (closed circles) and Ins^+^Glut2^LO^ (open circles) populations of β-cells isolated from neonatal mouse pancreas; and (**B**) Apelin and Aplnr expression in non-pregnant (NP) and pregnant mouse pancreas [gestational day (GD) 9–12 and 18]. Results are shown as fold increase compared to the geometric mean of the expression of housekeeping genes. Values represent mean ± SEM (n = 4–6). *p < 0.05 vs. Glut2^Hi^ in A, *p < 0.001 vs. NP in (**B**).

The findings from DNA microarray with respect to the apelinergic axis were validated using qPCR quantification of mRNA in fractions of Ins^+^Glut2^LO^ vs. Ins^+^Glut2^HI^ cells isolated from 7-day old mouse pancreata, relative to the expression of GAPDH and cyclophilin A. Levels of Apelin, Aplnr and Apela, were all expressed at significantly higher levels in Ins^+^Glut2^LO^ cells (Fig. 1A). Mean insulin-1 expression was lower in the Ins^+^Glut2^LO^ population compared with Ins^+^Glut2^HI^ cells, but not significantly so.

### Anatomical localization of the apelinergic system within the pancreas

**Figure 2:**
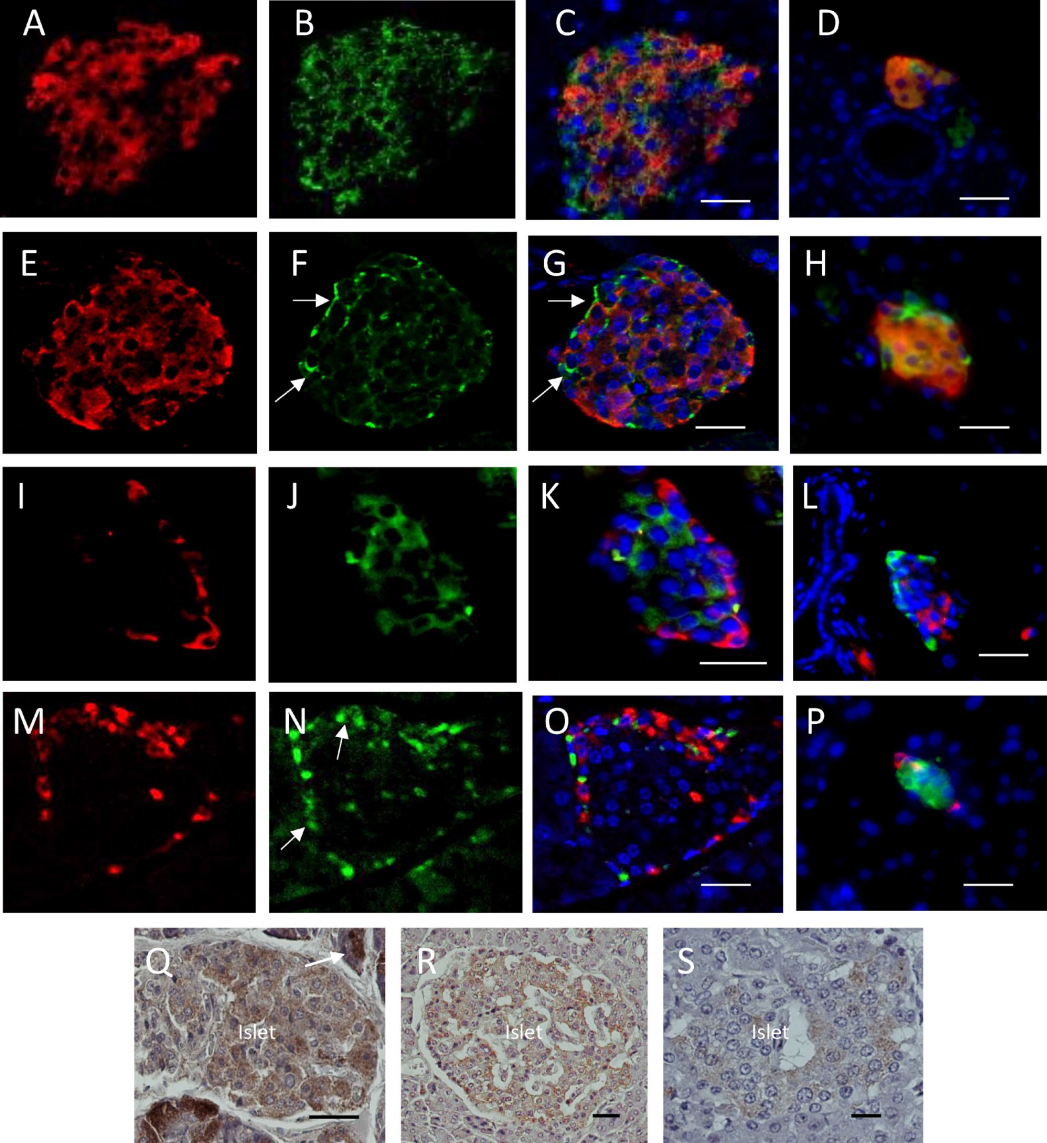
Immunohistochemical co-localization of insulin (**A** & **E**, red), glucagon (**I** & **M**, red), Apelin (**B** & **J**, green) and Aplnr (**F** & **N**, green) in adult mouse islets or extra-endocrine islet clusters (**D**,**H**,**L**,**P**). Merged images are shown for islets in (**C**), (**G**), (**K**) and (**O**) and for clusters. In merged images nuclei are shown stained with DAPI (blue). Arrows indicate the localization of Aplnr with the β-cell membranes. Immunohistochemical localization of Apelin in human pancreas is shown in panels (**Q**–**S**). Tissue donors were aged 4 months in (**Q**), 18 years in (**R**) and 41 years in (**S**). Apelin is localized to islet cells at all ages (Islet) and to acinar tissue (arrows) at 4 months. Bar represents 50 µm.

Immunohistochemical staining showed that Apelin was localized predominantly to a sub-population of insulin co-expressing β-cells in islets of Langerhans within adult mouse pancreata (Fig. 2A–C). Aplnr was also present and associated with the cell membrane in a sub-population of β-cells within islets that were mostly located towards the periphery of the islets (Fig. 2M–O). The distribution of Aplnr on the cell membranes was strongly punctate with less intense staining being present within the cytoplasm. Extensive co-localization of Apelin and/or Aplnr with insulin was seen in the small, extra-islet endocrine cell clusters (Fig. 2D,H). When glucagon was localized as a marker of α-cells only occasional co-localization was observed with either Apelin or Aplnr within islets (Fig. 2I–K,M–O) or clusters (Fig. 2L,P). A similar distribution of Apelin and Aplnr was also seen in islets within pancreata from neonatal mice (Supplementary Fig. 1). In addition to localization to β-cells Aplnr immunostaing was also observed to be associated with some vascular endothelial cells within the core of the islets. We also examined the presence of Apelin in human pancreas from a range of donor ages between early childhood and adulthood. Apelin was localized to islet endocrine cells with the intensity of staining decreasing with age. Apelin was also located within a sub-population of acinar cells towards the periphery of the growing pancreas at early ages but was less apparent in adulthood (Fig. 2Q–S). Images from an age range of additional donors are shown on Supplementary Fig. 2).

**Figure 3:**
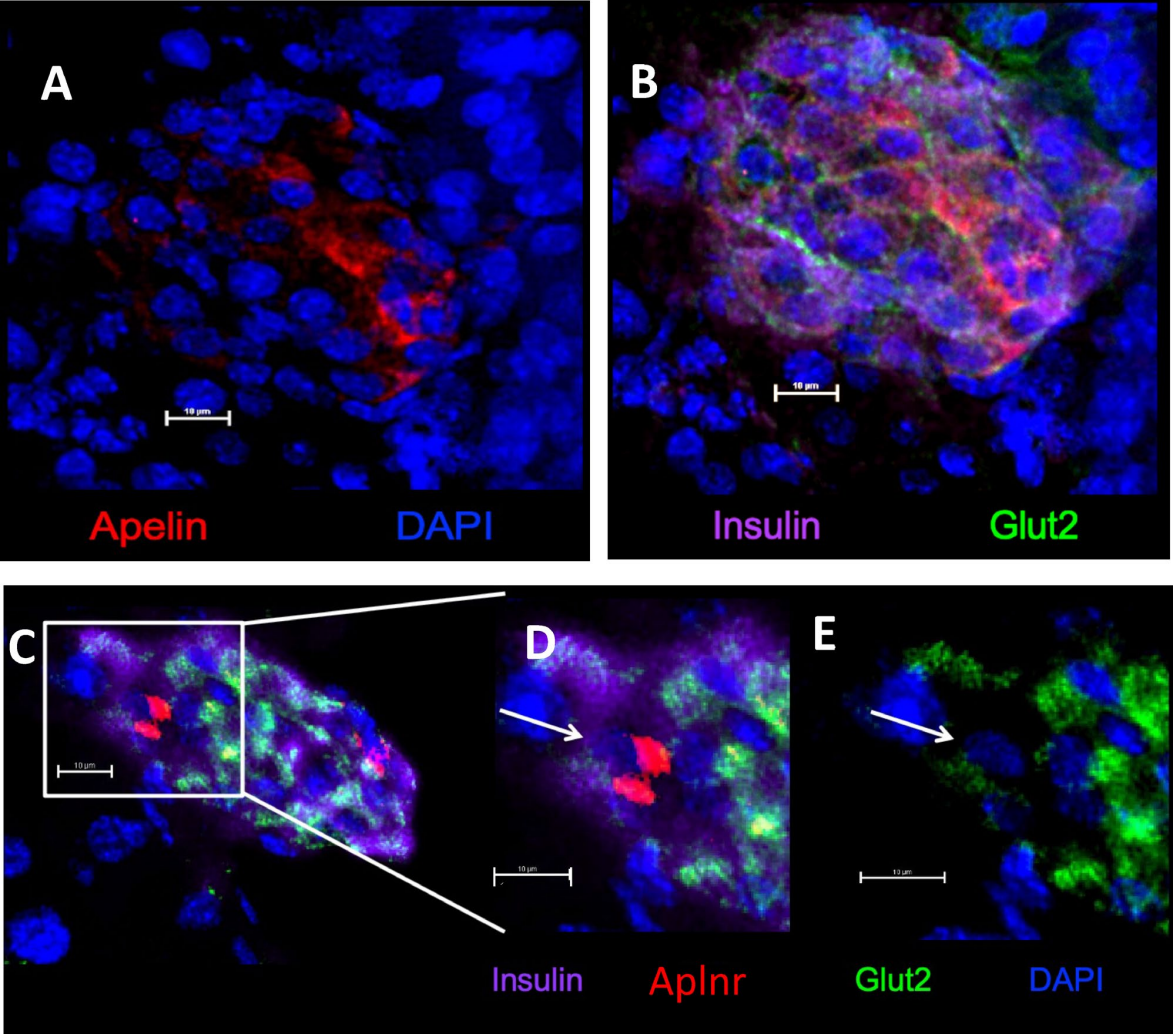
Immunohistochemical localization of Apelin alone (**A**), and in a merged image with insulin and Glut2 (**B**) in adult mouse pancreatic islets. Localization of insulin, Aplnr and Glut2 is shown in (**C**) with an expanded view of co-localization of Aplnr to Ins^+^Glut2^LO^ cells in (**D**) (arrows). The localization of Glut2 alone in the same section is shown in (**E**). Nuclei are counter-stained with DAPI (blue). Bar represents 10 µm.

We further defined the sub-population of β-cells in mouse islets that contained Apelin and Aplnr by co-staining with Glut2. Apelin predominantly co-localized to β-cells that also showed strong Glut2 staining (Fig. 3A,B). In contrast, Aplnr was largely confined to β-cells in the islet mantle that showed no Glut2 staining (Fig. 3C–E).

### Changes in the pancreatic apelinergic system during pregnancy

**Figure 4:**
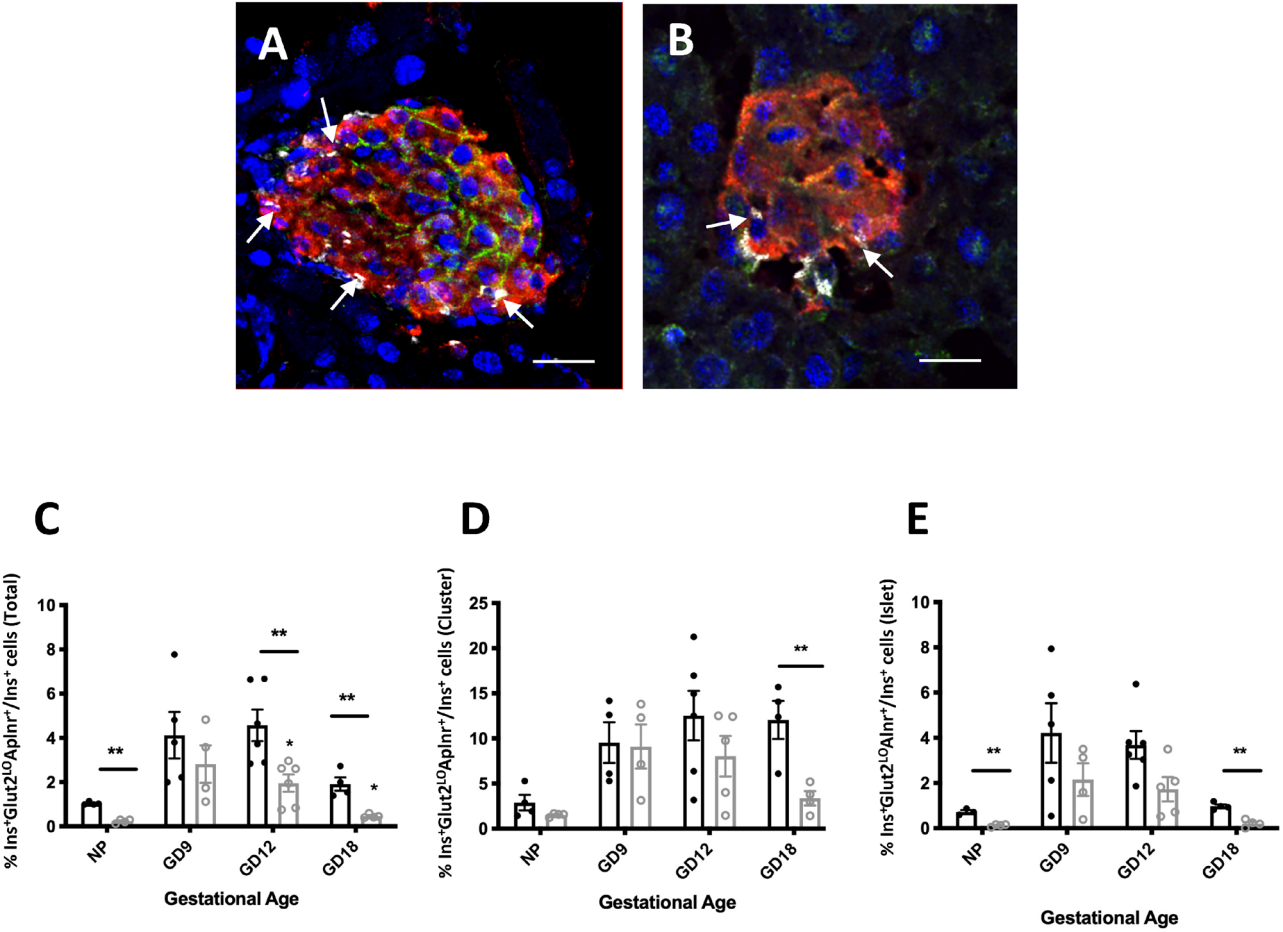
Immunohistochemical localization of Aplnr (white), insulin (red), Glut 2 (green) and cell nuclei (DAPI, blue) in islets from pregnant mice at GD 12 exposed in early life to control (**A**) or LP (**B**) diet. Co-localization of Aplnr to Ins^+^ Glut2^LO^ cells is indicated by arrows. Bar represents 80 µm in (**A**) and 50 µm in (**B**). The percent Ins^+^ Glut2^LO^ Aplnr^+^ cells relative to all Ins^+^ cells is shown for total pancreas (**C**), extra-islet clusters (**D**) or within islets (**E**) for control (closed circles, black bars) or LP pregnancies (open circles, grey bars). Values represent mean ± SEM (n = 4–6) in non-pregnant females (NP) or at gestational day (GD) 9, 12 or 18. *p < 0.05, **p < 0.001 vs. control.

The expression of Aplnr and its ligands were quantified by qPCR in isolated islets from pregnant mice relative to non-pregnant animals. Apelin mRNA levels did not differ between pregnant and non-pregnant mice, but expression of Aplnr significantly declined in late pregnancy (Fig. 1B). The presence of Apela mRNA was not detectable. However, changes in apelinergic gene expression in minority cell populations such as Ins^+^Glut2^LO^ cells might be difficult to detect within whole islets. Therefore, we examined changes in the number of Aplnr-immunoreactive cells at various gestational ages compared with non-pregnant, age-matched mice. During pregnancy, as in non-pregnant mice, Aplnr was predominantly localized to Ins^+^Glut2^LO^ cells (Fig. 4A) and the abundance of such cells significantly increased at GD 9 and 12 (p < 0.01) before decreasing at GD 18, when considering whole pancreas (Fig. 4C). When the location of Ins^+^Glut2^LO^Aplnr^+^ cells was separated into islet or extra-islet endocrine cluster compartments, a similar ontological profile was seen for islets (Fig. 4E), however, the frequency of these cells was two- to three-fold higher in clusters and did not decline in later gestation (Fig. 4D).

We utilized a mouse model of glucose intolerance in pregnancy where female offspring of dams exposed to a low protein (LP) diet between conception and weaning have a lower BCM when pregnant, as compared to offspring of control-fed dams^21^. We examined the abundance of Ins^+^Glut2^LO^Aplnr^+^ cells in pregnant mice exposed to the maternal LP diet in early life. The abundance of such cells was significantly reduced in pregnant mouse pancreata from LP-exposed mice at GD 12 and 18 compared to control-fed animals, although a pregnancy-associated increase in their number still occurred (Fig. 4B,C). A similar pattern was seen when data was separated into islet and extra-islet cluster compartments (Fig. 4D,E). Of note, these differences may originate prior to pregnancy as the abundance of Ins^+^Glut2^LO^Aplnr^+^ cells was significantly lower in the pancreas of non-pregnant mice that previously received the LP diet.

To determine if this decrease in abundance of Ins^+^Glut2^LO^Aplnr^+^ cells in pancreata from glucose intolerant pregnant mice reflected a general decrease of Ins^+^Glut2^LO^ cells related to LP diet we compared the percentage of Ins^+^Glut2^LO^ cells relative to all Ins^+^ cells at each gestational day. For both control and LP pregnancies, Ins^+^Glut2^LO^ cell presence significantly deceased after GD 9 in whole pancreas and when considering clusters alone but did not differ with prior diet (Table 2). Therefore, the reduced presence of Aplnr immunoreactivity in Ins^+^Glut2^LO^ cells in LP vs. control pregnancies was not due to an associated change in Ins^+^Glut2^LO^ cell abundance.

**Table 2:** Percentage of Ins^+^Glut2^LO^ cells relative to total insulin immunoreactive β-cells in histological sections of non-pregnant (NP) and pregnant mouse pancreas (GD 9–18) previously exposed in early life to control or low protein (LP) diet.

	Whole pancreas		Extra-islet endocrine clusters	
	Control diet	LP diet	Control diet	LP diet
NP	1.13 ± 0.11	0.89 ± 0.21	6.08 ± 0.70	4.12 ± 0.61
GD 9	1.32 ± 0.08	1.05 ± 0.14	9.49 ± 1.38*	8.46 ± 1.76
GD 12	0.89 ± 0.21	0.51 ± 0.05^†^	3.69 ± 0.56^†^	5.65 ± 1.88^†^
GD 18	0.42 ± 0.08^†,#^	0.50 ± 0.07^†^	4.34 ± 0.92^†^	4.13 ± 0.52^†^

Values show mean ± SEM (n = 4–6) for percentage of Ins^+^Glut2^LO^ cells compared to all insulin immunoreactive cells for entire pancreas sections and for the population of extra-islet endocrine clusters alone. *p < 0.05 vs, NP, ^†^p < 0.05 vs. GD9, ^#^p < 0.01 vs. NP, one way analysis of variance. Comparisons by two way analysis of variance between control and LP diet showed no significant differences between mean values for either whole pancreas or clusters.

### Biological actions of Apelin and Apela on β-cells

**Figure 5:**
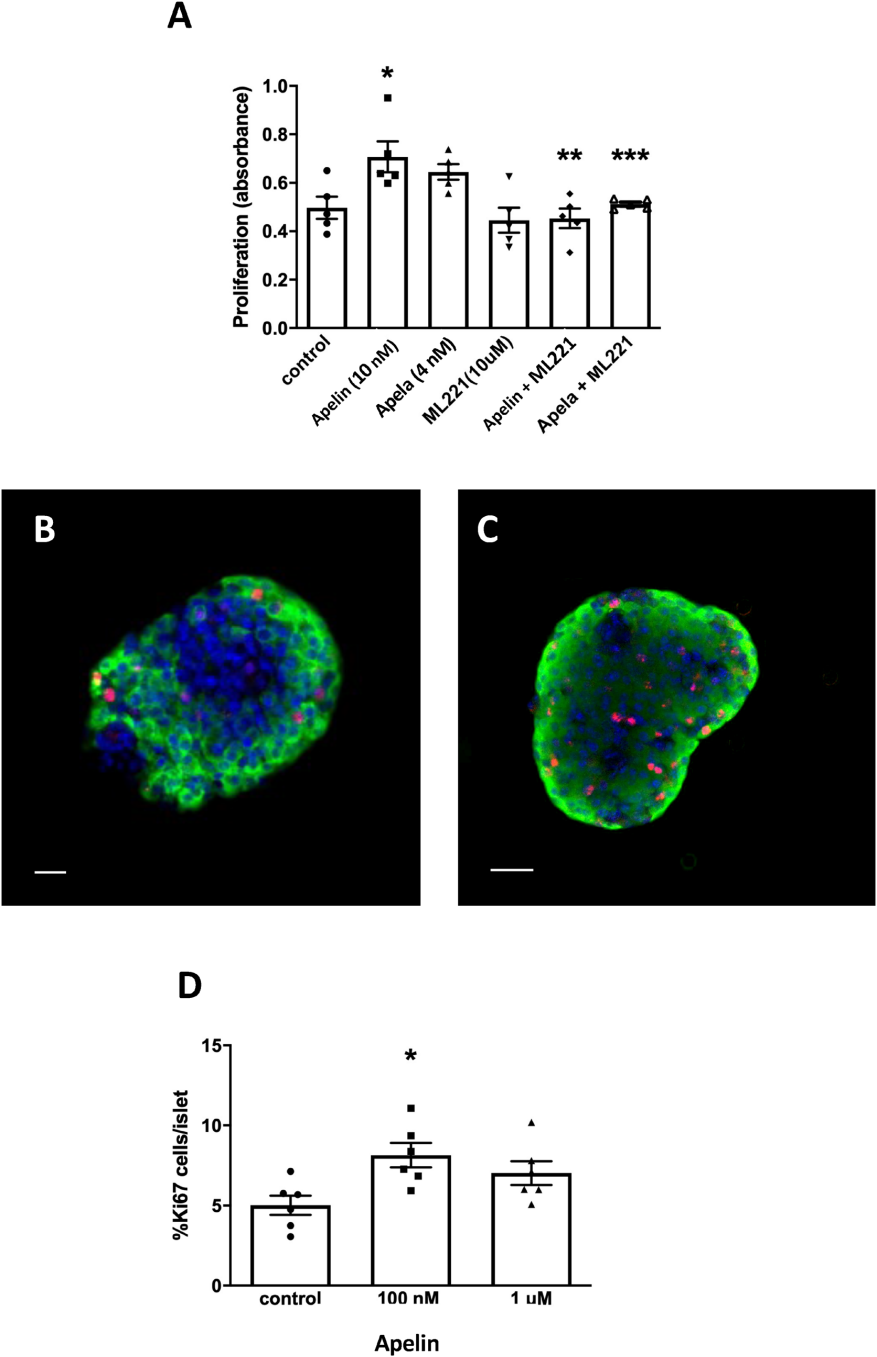
(**A**) Proliferation of INS1E cells measured by MTT assay in the presence or absence of Apelin (10 nM) or Apela (4 nM) with or without the addition of the Aplnr inhibitor ML221 (10 µM). Values represent mean ± SEM (n = 4–6). *p < 0.05 vs. control, **p < 0.01, ***p < 0.001 vs. Apelin or Apela alone. Cell proliferation of β-cells visualized by fluorescence immunohistochemistry for insulin (green) and Ki67 (red) in islets isolated from neonatal mice incubated without (**B**) or with Apelin (100 nM, **C**). Bar represents 50 µm. The percentage of Ki67-positive β-cells was quantified (**D**) in control culture and in the presence of 100 nM or 1 μM apelin. Values represent mean ± SEM (n = 5–6 replicates per experiment). *p < 0.05 vs. control.

A 4,6-diamidino-2 phenylindole, dihydrochloride (MTT) cell proliferation assay was used to examine the actions of Apelin and Apela on INS1E β-cells. Apelin significantly increased cell proliferation at 10 nM (Fig. 5A), but not at lower or higher concentrations. Apela did not significantly increase cell proliferation at 4 nM or greater concentrations relative to controls. Co-incubation with the specific Aplnr antagonist, ML221 significantly negated the proliferative effects of both Apelin and Apela (Fig. 5A) while having no detrimental effect alone. This suggests that any endogenous release of Apelin or Apela from INS1E cells was minimal. We also examined the ability of Apelin to increase β-cell DNA synthesis within islets isolated from neonatal mice. Immunohistochemistry was performed on whole islets to co-localize insulin and Ki67 as a measure of β-cells in G1 and S phase of cell replication (Fig. 5B,C). Apelin significantly increased DNA synthesis in β-cells, as measured by co-localization of insulin and Ki67, at 100 nM but not at greater concentrations (Fig. 5D).

**Figure 6:**
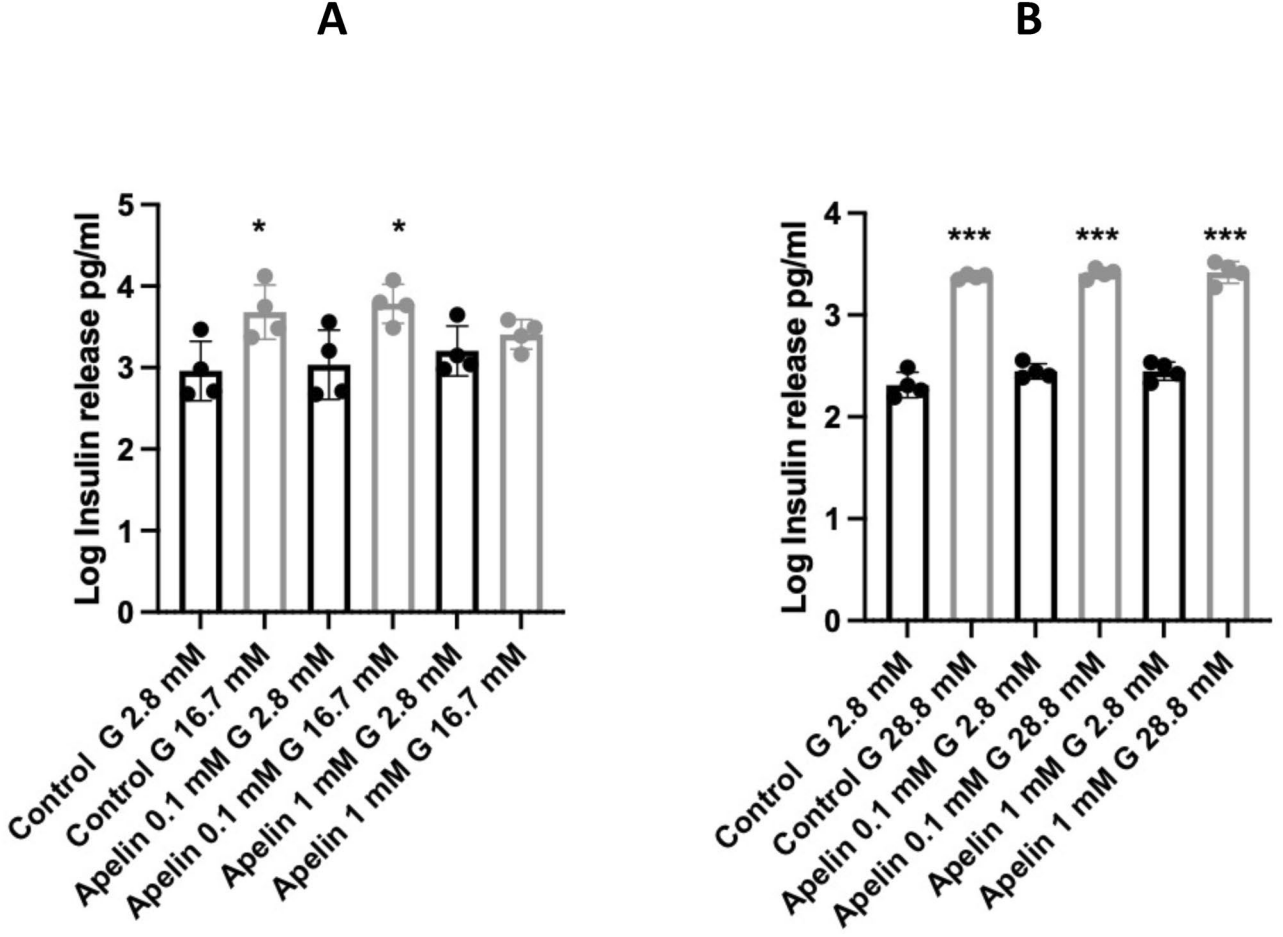
Insulin release (log pg/ml) from (**A**) isolated neonatal mouse islets and (**B**) INS1E cells in the presence of basal glucose (2.8 mM, black bars) or a stimulating concentration (16.7 mM glucose for islets or 28.8 mM for INS1E cells; grey bars), with or without Apelin at 0.1 mM or 1 mM concentrations. Values represent mean ± SEM (n = 4). *p < 0.05, ***p < 0.001 vs. 2.8 mM glucose.

Similarly, the actions of Apelin on GSIS were investigated in both isolated islets from neonatal mice and in INS1E cells (Fig. 6). Incubation in the presence of 16.7 mM glucose (islets) or 28.8 mM glucose (INS1E cells) resulted in a significant increase in insulin secretion (expressed as log values) relative to control cultures containing 2.8 mM glucose. However, the addition of Apelin at 0.1 or 1 mM did not modify insulin release at either basal or stimulating glucose concentrations. When expressed as a fold increase in insulin release between the lower and higher glucose concentrations for islets mean values for control cultures were 6.4 ± 2.1, 7.2 ± 1.7 for Apelin at 0.1 mM and 2.6 ± 0.4 at 1 mM Apelin. Fold increase values for INS1E cell cultures were 10.2 ± 1.4, 8.8 ± 0.3 for Apelin at 0.1 mM and 9.2 ± 0.2 at 1 mM Apelin. Thus, the delta changes in glucose-stimulated insulin release were not significantly altered by Apelin.

### The placental apelinergic axis

**Figure 7:**
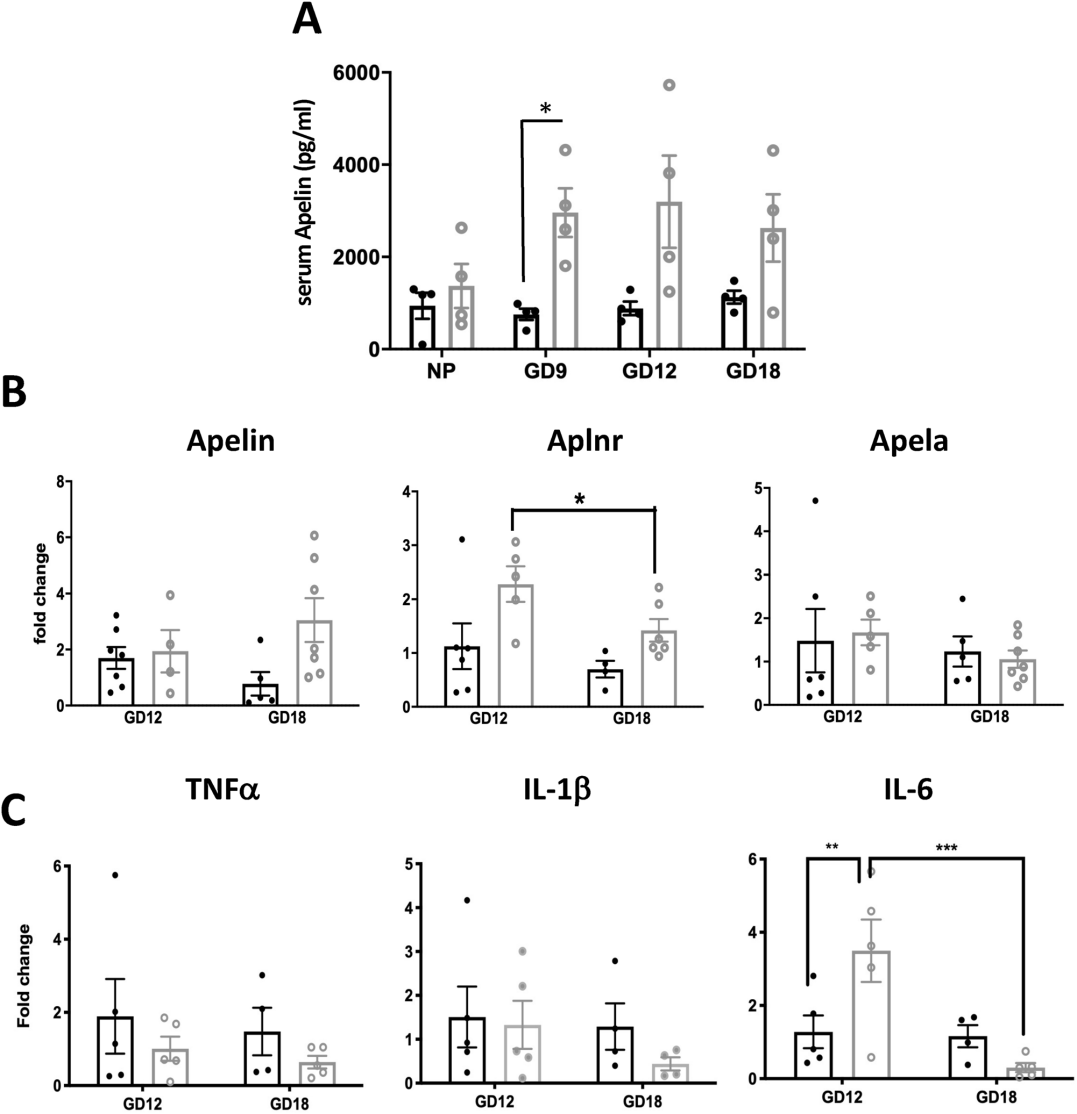
(**A**) Serum levels of Apelin detectable in non-pregnant female mouse serum (NP) and at gestational days (GD) 9, 12 and 18 in animal receiving control (closed circles, black bars) or LP diet (open circles, grey bars) in early life. (**B**) Expression levels of mRNA for Apelin, Aplnr and Apela in placenta from (**C**) (black bars) or LP-exposed (grey bars) pregnant mice on GD 12 and 18; and (**C**) expression levels of TNFα, IL-1β and IL-6 in placenta at the same gestational ages. Values represent mean ± SEM (n = 4–6). *p < 0.05, **p < 0.01, ***p < 0.001 vs. control or between days.

The mitogenic effects of Apelin on β-cells coupled with the increased BCM that occurs during pregnancy could be linked to a placental production of Apelin or Apela. We found no significant change in maternal serum levels of Apelin through gestation during normal pregnancy (Fig. 7A). Maternal Apelin levels in dams who were exposed to the LP diet in early life were significantly greater than those in control-fed animals at GD 9, but not at other times. We also quantified mRNA levels for Apelin, Apela and Aplnr in placental tissues from mice at GD12 and 18 (Fig. 7B). All three proteins were expressed, but levels did not change between GD 12 and 18 in control pregnancies. In glucose intolerant pregnancies the levels of placental Aplnr expression were higher at GD 12 than at GD 18, but did not differ with diet. Expression levels of Apelin and Apela also did not differ with diet.

Lastly, since GDM is characterized by an enhanced pro-inflammatory environment with elevated levels of pro-inflammatory cytokines that might precipitate β-cell dysfunction, we also quantified mRNA levels of TNFα, IL-1β and IL-6 in placentae from control and LP pregnancies. IL-6 was more highly expressed in LP pregnancies when compared to controls at GD 12, but not at GD 18, whilst TNFα and IL-1β levels did not differ with prior diet (Fig. 7C).

## Discussion

Our findings confirm that Apelin, Apela and Aplnr are preferentially expressed within the mouse pancreatic β-cell population from neonatal life until adulthood, and that Aplnr is predominantly expressed within Ins^+^Glut2^LO^ β-cells most abundant in the periphery of the islets of Langerhans. The abundance of Aplnr-stained Ins^+^Glut2^LO^ β-cells increased three- to four-fold during pregnancy being maximal at gestational days 9–12 and were significantly reduced in glucose intolerant mice. The likelihood that increased signaling through the apelinergic axis in pancreas contributes to the increased β-cell proliferation seen during pregnancy is supported by the mitogenic effects of Apelin in isolated mouse islets and INS1E cells.

These findings reinforce previous evidence^19,20,42^ that Ins^+^Glut2^LO^ pancreatic endocrine cells have a different distribution and gene expression profile compared to Ins^+^Glut2^HI^ cells. Specifically, the Ins^+^Glut2^LO^ cells more highly express a wide range of genes associated with β-cell lineage commitment (such as delta like non-canonical notch ligand 1^43^ and fatty acid binding protein-4^44^), proliferation, extracellular matrix remodeling, and angiogenesis. This further supports the hypothesis that Ins^+^Glut2^LO^ cells are a source of new β-cells given the intimate relationship between the islet microvasculature and BCM, and the role of the extracellular matrix in defining the paracrine signaling that supports the proliferation of both β-cells and endothelial cells^45,46^. Many of the genes differentially expressed in Ins^+^Glut2^LO^ cells have also been reported as being selectively up-regulated in mouse islets during pregnancy^47^, supporting the likelihood that Ins^+^Glut2^LO^ cells contribute to the increase in BCM observed during mouse pregnancy.

The relatively high expression of apelinergic system genes, Apelin, Apela, and Aplnr, within Ins^+^Glut2^LO^ cells was confirmed by qPCR for isolated islets relative to Ins^+^Glut2^HI^ cells while immunohistochemistry showed that Apelin was preferentially co-localized within most β-cells in mouse and human pancreas. The semi-quantitative nature of immunohistochemistry may explain why staining for Apelin was not noticeably different between Ins^+^Glut2^HI^ and Ins^+^Glut2^LO^ β-cells despite mRNA expression being significantly greater in the latter. The Ins^+^Glut2^LO^ cells were preferentially localized in the periphery of the islets, as we described previously^48^ which may represent a ‘niche’ for new β-cell development from progenitor cells^49^. Aplnr was more abundantly expressed in Ins^+^Glut2^LO^ than Glut2^HI^ cells and the peptide was similarly preferentially localized by immunohistochemistry, although localization was also seen in a minority of α-cells as described before^37^. Additionally, Aplnr was localized to some small cells in the core of the islet with the morphology of endothelial cells. This is consistent with the reported ability of Apelin to promote endothelial cell differentiation^50^ Apelin was also present in the acinar cells around the periphery of the human pancreas in neonatal subjects, but not adults. In rodent species new pancreatic lobes continue to develop in early postnatal life with proliferation of acinar cells^51^. If pancreatic lobes continue to be formed postnatally in human then Apelin expression could possibly contribute to this process.

Aplnr has been previously linked to the β-cell generation^38^. However, this action could be indirect due to the ability of Apelin to promote angiogenesis through the maturation of endothelial cell progenitor cells^52^. Our findings suggest that Apelin directly promotes β-cell DNA synthesis as seen in both isolated islets and INS1E cells, and the use of a selective Aplnr antagonist demonstrated that the actions were mediated by the Aplnr receptor. Both Apelin and Apela have been shown to activate the PI3K/AKT/mTORC1 signaling pathways, which are potent regulators of proliferation and facilitate a reduction in apoptosis^53^.

During mouse pregnancy pancreatic Ins^+^Glut2^LO^ cells are highly proliferative at mid-gestation but this declines in late gestation, possibly through their maturation into functional β-cells^20^. A similar pattern was seen here during pregnancy for the number of Ins^+^Glut2^LO^ cells expressing Aplnr, suggesting that the apelinergic system may contribute to the increased BCM. In support of this hypothesis a long-acting Apelin analogue was shown to increase β-cell area within islets following administration of streptozotocin, or following a high fat diet, in mice^54^. The relative abundance of Ins^+^Glut2^LO^Aplnr^+^ cells was significantly reduced in a mouse model of gestational hyperglycemia characterized by a lower BCM further suggesting a causal relationship.

We found no effect of Apelin on GSIS in vitro from INS1E cells or from isolated mouse islets. Previous reports using the same cell line, isolated islets or administration in vivo have been inconsistent^37,55,56^. However, Apelin has several metabolic actions including the inhibition of lipolysis, regulation of glucose uptake and fatty acid oxidation, and increased mitochondrial bioactivity^57^. Thus, glucose homeostatic actions in vivo may be a combination of both direct and indirect effects on metabolic tissues. The biological actions of Apelin might also differ between molecular forms. Apelin is synthesized as a 77 amino acid prepropeptide that can be differentially cleaved in a tissue-specific manner at the C-terminal to yield peptides of 35, 17 or 13 amino acids, each with different potencies with respect to Aplnr signaling^58^. In our studies we utilized the shorter, Apelin-13 form.

The short biological half-life of Apelin means that circulating levels are low (0.02–0.05 pmol/mL in rats)^59^, implying that locally produced Apelin is likely of most relevance to the control of BCM. However, this may differ during pregnancy when maternal levels increase due to the release of Apelin from the placental syncytiotrophoblast, as reported in humans^28^. We could not confirm an increasing gestational presence of Apelin in mice, although circulating levels were higher in both non-pregnant and pregnant mice (approximately 1 nM) than those described in women. However, mRNAs for Apelin, Apela and Aplnr were each expressed in mouse placenta. In hyperglycemic mouse pregnancies Apelin levels only differed from values in control pregnancies in mid-gestation and the placental expression of Apelin, Apela, and Aplnr did not differ. However, cellular stress may have been occurring in placentae from glucose intolerant pregnant mice related to a selective increase in IL-6 expression, as was also observed in human gestational diabetes^60^. Interestingly, incubation of human syncytiotrophoblast cells with increasing concentrations of human Apelin decreased the release of human placental lactogen^61^, a major trophic factor for the expansion of BCM during pregnancy^8–11^. Notably, in human pregnancies with GDM, maternal levels of Apelin were relatively increased in the second trimester, as was observed in the present studies for hyperglycemic mouse pregnancies, whilst levels of Apela were decreased^62^. The relationship between placental expression of Apelin and BCM during pregnancy is therefore likely to be complex.

In summary, our studies demonstrate the presence of Apelin in pancreatic β-cells throughout mouse pregnancy and show that Apelin exerts mitogenic effects on β-cells through the Aplnr receptor. Aplnr was preferentially localized to pancreatic Ins^+^Glut2^LO^ cells during pregnancy, and the proportion of such cells immunopositive for Aplnr was decreased in glucose intolerant pregnancy. Thus, we speculate that the apelinergic axis contributes to the increased BCM of pregnancy.

## Materials and methods

### Animals

A total of 180 C57B6/6J mice (Charles River Laboratories, Wilmington, MA, USA) were used in the studies that generated the data reported. Animals received standard mouse chow and water ad libitum unless otherwise indicated. The studies were compliant with the ARRIVE guidelines both in the design and reporting of the findings. All animal procedures received animal ethics committee approval from Western University, Canada and were undertaken with adherence with the standard operating practices established by Western University and in agreement with published guidelines of the Canadian Council for Animal Care.

Time-mated pregnant mice aged 42–50 days were allowed to deliver and neonatal animals were euthanized by decapitation at 7 days of age before removal of the pancreas for enzymatic dispersal to single cell suspensions prior to fluorescence-activated cell sorting (FACS). The rationale for using neonatal mice was that the number of Ins^+^Glut2^LO^ cells is greatest in early life^42^. Pancreata were also collected from pregnant mice at gestational days (GD) 9 and 12 and age-matched non-pregnant females following euthanasia by CO_2_ asphyxia prior to isolation of the islets of Langerhans.

In separate experiments female mice were time-mated and randomly allocated when pregnant to either a C diet (20% protein, Bioserv, NJ, USA) or a low protein (LP) isocaloric diet (8% protein with a balance of calories from sucrose, Bioserv)^21^. The respective diets were maintained throughout gestation until weaning (post-natal day 21), at which point the offspring (F1) were transferred to the C-diet. At age 42–50 days, F1 female mice previously exposed to LP or C diets were randomized into pregnant or non-pregnant groups. Those in the pregnancy group were time-mated with C diet-fed C57BL/6J males. LP- and C-exposed pregnant F1 mice were euthanized on either GD 9, 12 or 18. Pancreata and placentae were then removed, weighed, and either fixed in 4% paraformaldehyde for histology, or placed into RNA later (QIAGEN, Hilden, Germany) prior to storage at – 20 °C for future RNA isolation. Blood was collected via cardiac puncture after death and serum separated to quantify circulating apelin.

Additional details of animal care and analytical methods are provided as Supplementary Information.

### Fluorescence activated cell sorting (FACS) and DNA microarray analysis

Dispersed cells from whole pancreata of 7-day-old mice were subjected to FACS as described previously^19^. Cells fractions were separated based on the binding of antibodies against GPm6a (a cell surface marker specific for mouse β-cells^63^) and Glut 2 to create Ins^+^Glut2^HI^ or Ins^+^Glut2^LO^ fractions. Using the RNeasy Plus Mini kit (QIAGEN), total RNA was extracted and purified from each cell pool and DNA microarray analysis performed at the London Regional Genomics Centre, Western University, London, ON, Canada (Mouse Genome 430 2.0 (MOE430 2.0) array, Affymetrix, Santa Clara, CA, USA). All procedures, including cRNA synthesis, labelling, and hybridization were performed as described in the Affymetrix Technical Analysis Manual. The GeneChips were scanned with the Affymetrix GeneChip Scanner 3000 and probe level data from the .CEL files were analysed using Partek Genomics Suite v6.5 (Partek, St. Louis, MO, USA). Probes were imported and summarized using multi-array averaging and ANOVA was used to determine fold changes. Only those genes with a tenfold or greater difference in expression between Ins^+^Glut2^HI^ or Ins^+^Glut2^LO^ cell fractions were considered further.

### Quantitative polymerase chain reaction (qPCR)

RNA was extracted from Ins^+^Glut2^HI^ or Ins^+^Glut2^LO^ cell fractions from 7 day-old neonatal mouse pancreata, isolated islets of Langerhans from pregnant mouse pancreata, and from mouse placentae and stored at – 80 °C. Quantitative PCR was performed on a QuantStudio5 Real-time PCR System (Applied Biosystems, Waltham, MA, USA) using TaqMan primers for Apelin, Apela, Aplnr, insulin, TNF-α, IL-1ß, IL-6 and for the control genes, cyclophilin A (cycloA) and glyceraldehyde-3-phosphate dehydrogenase (GAPDH) to quantify relative gene expression using the ∆∆ cycle threshold (C_T_) method. Relative gene expression was calculated as fold change compared to the geometric mean of the housekeeping genes GAPDH and cyclophilin A.

### Immunohistochemistry

At least two longitudinal cryosections (7 μm) were examined from each mouse pancreas with an interval greater than 100 μm between each. Immunofluorescence histochemistry was performed to localize Apelin, Aplnr, insulin, glucagon, somatostatin and Glut2 as described previously^20^. Full details of antibody sources and dilutions are provided in the Supplementary Methods. Formalin-fixed, paraffin embedded sections of non-diabetic human pancreas were obtained from the Department of Pathology and Laboratory Medicine, Western University with institutional approval from the Western University Human Research Ethics Board. All methods were performed in accordance with the guidelines and regulations governing the use of human pathological samples by Western University through the research ethics board.

Immunohistochemical staining for Apelin was performed using diaminobenzidine (DAB) as the chromogen. Tissue sections were de-identified and the histology quantified using a Nikon Eclipse TS2R inverted microscope (Nikon, Minato, Tokyo, Japan) with the program NIS elements (Nikon, Minato). Images were captured and analyzed using cell counter on ImageJ software. Every insulin, Aplnr, or Glut2-expressing cell was imaged for each section and for each animal. In this study, an “islet” was considered to contain six or more β-cells, and an extra-islet endocrine “cluster” containing 1–5 β-cells^19^.

### Isolated islet and INS1E cell culture

Pancreata from neonatal or pregnant mice were digested with collagenase V and islets separated using a Dextran density gradient consisting of 27, 23 and 11% concentrations and collected from the 23/11% interface. Islets were incubated for 24 h and allocated the following day into 6-well ultra-low attachment multiwell plate (Falcon, VWR International) in RPMI medium for 48 h, with and without Pyr-Apelin 13 (100 nM, 1 μM; Sigma. Following exposure to Apelin, islets (approximately 20 islets/treatment) were hand-picked and allowed to affix to glass-bottom dishes (MatTek Life Sciences, Ashland, MA, USA) pre-adsorbed with diluted Cell-Tak adhesive (BD Biosciences), fixed in 4% paraformaldehyde for 30 min at room temperature and stored at 4 °C in phosphate buffered saline (PBS). Immunofluorescent staining for insulin and Ki67 was performed on whole islets to assess the percentage of β-cells undergoing DNA synthesis.

Z-stack images were collected from control or Apelin-treated islets using confocal microscopy (Nikon A1R, Nikon Canada, Mississauga, ON, Canada) with an average of 26 images per stack. Four to six randomly selected images per islet (20–25 islets/treatment) were analyzed using the cell counter on ImageJ software and the percentage of Ki67^+^ cells relative to insulin^+^ cells was calculated.

An MTT assay was used to determine the effects of Apelin or Apela on the proliferation of INS1E cells (gifted by Dr. D. Kilkenny, University of Toronto). Culture medium contained Apelin (10 nM) or Apela (4 nM) with or without the specific Aplnr antagonist, ML221 (10 µM; BioTechne, Minneapolis, MN, USA). Spectrophotometric absorbance of the purple formazan crystal product was measured using a microplate reader at an absorbance wavelength of 590 nm.

Isolated islets (approximately 20 islets/well) and INS1E cell cultures (approximately 70% confluency) were also incubated in the presence or absence of Apelin (100 nM or 1 μM) for 48 h prior to incubation with either low (2.8 mM) or high (islets 16.7 mM, INS1E cells 28.8 mM) glucose for 90 min at 37 °C. Conditioned culture medium was removed and insulin release determined using an Ultra-Sensitive Mouse Insulin ELISA kit (Crystal Chem, Elk Grove Village, IL, USA).

### Apelin measurement

A competitive enzyme-linked immunosorbent assay (ELISA) was used to quantify Apelin in pregnant and non-pregnant mouse serum using the manufacturer’s instructions (NBP2-68235, Novus Biologicals, Centennial, CO, USA). Data was then collected using an iMark Plate Reader (Bio-Rad) and analyzed using Microplate Manager Software (Bio-Rad).

### Statistical analysis

A sample size of 4–6 animals per variable was utilized based on achieving a statistically significant difference (p < 0.05) with an expected standard deviation around mean values of 15% or less for pancreas immunohistochemistry, based on previous studies^19,20^. Where duplicate tissue sections from the same pancreas gave discordant values two further sections were examined. DNA microarray analysis was repeated twice. Statistics were conducted using GraphPad Prism software and data are presented as mean ± SEM. The data were analyzed using either a Student’s unpaired t-test, a one-way ANOVA or a two-way ANOVA and a Tukey post-hoc test was conducted to compare study groups at different experimental time points. Insulin values were log transformed before analysis.

## Supplementary Information

Supplementary Figure 1.

Supplementary Figure 2.

Supplementary Information.

Supplementary Table 1.

## Data Availability

The datasets generated and analysed during the current study are available from the corresponding author on reasonable request.
