# Supplementary material for: Ontology of the apelinergic system in mouse pancreas during pregnancy and relationship with β-cell mass
Source: Sci Rep. 2021 Jul 29;11:15475. doi: 10.1038/s41598-021-94725-0 (PMC8322410; doi:10.1038/s41598-021-94725-0)
Supplement: Supplementary file 1 — Supplementary Figure 1. [file 41598_2021_94725_MOESM1_ESM.pdf]

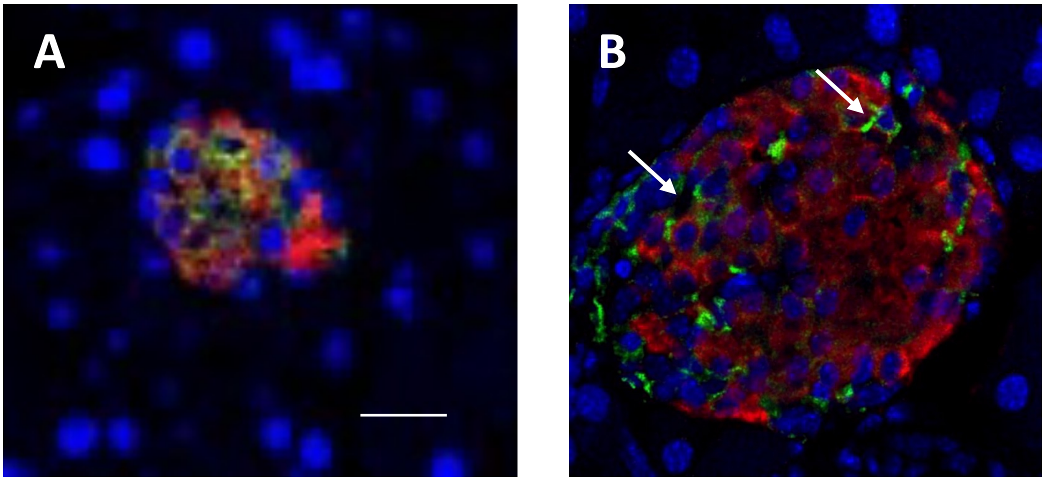

Supplementary Figure 1

Legend

Immunohistochemical co-localization of insulin (red) with A) Apelin and B) Aplnr (green) in neonatal mouse islets. Arrows indicate the localization of Aplnr with the β-cell membranes. Nuclei were stained with DAPI (blue). Bar represents 50 μm.
