# Supplementary material for: Ontology of the apelinergic system in mouse pancreas during pregnancy and relationship with β-cell mass
Source: Sci Rep. 2021 Jul 29;11:15475. doi: 10.1038/s41598-021-94725-0 (PMC8322410; doi:10.1038/s41598-021-94725-0)
Supplement: Supplementary file 2 — Supplementary Figure 2. [file 41598_2021_94725_MOESM2_ESM.pdf]

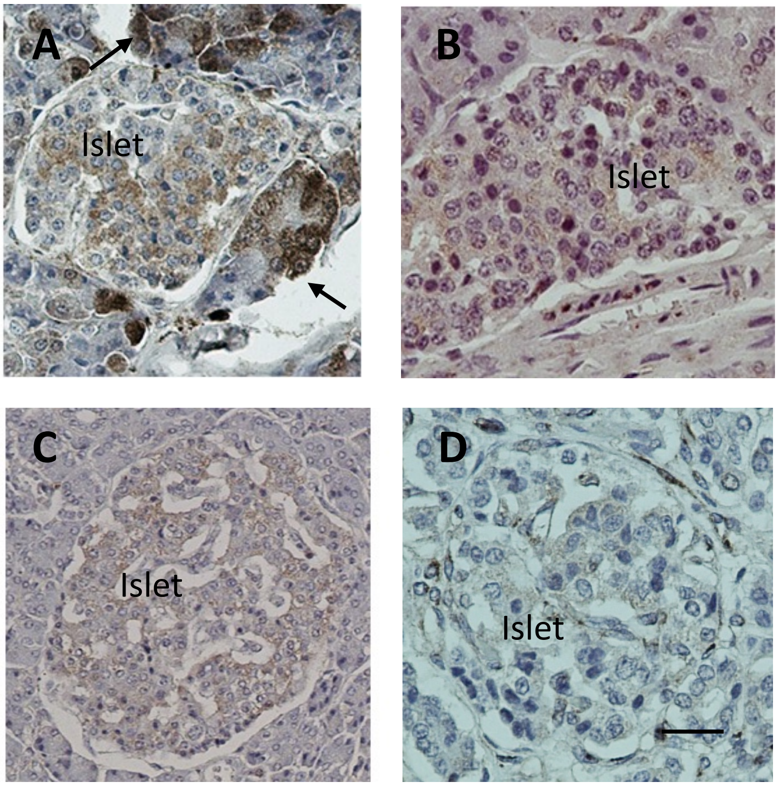

Supplementary Figure 2

Legend

Immunohistochemical localization of Apelin in human pancreas for donors aged A) 4 weeks, B) 2 years, C) 18 years and D) 63 years. Apelin is localized to islet cells at all ages (Islet) and to peripheral acinar tissue (arrow) in early life. Bar represents 100 μm.
