# Supplementary material for: Ontology of the apelinergic system in mouse pancreas during pregnancy and relationship with β-cell mass
Source: Sci Rep. 2021 Jul 29;11:15475. doi: 10.1038/s41598-021-94725-0 (PMC8322410; doi:10.1038/s41598-021-94725-0)
Supplement: Supplementary file 3 — Supplementary Information. [file 41598_2021_94725_MOESM3_ESM.docx]

SUPPLEMENTARY MATERIAL

Ontology of the apelinergic system in mouse pancreas during pregnancy and relationship with β-cell mass

Brenda Strutt, Sandra Szlapinski, Thineesha Gnaneswaran, Sarah Donegan, Jessica Hill, Jamie Bennett and David J. Hill

SUPPLEMENTARY METHODS

Animals: C57B6/6J mice were housed at the Lawson Health Research Institute, London, ON, Canada under a 12-hour light/ dark cycle. Animals were housed four per cage with environmental enrichment and received standard mouse chow (7% simple sugars, 3% fat, 50% polysaccharide, 15% protein (w/w), energy 3.5 kcal/g) and water *ad libitum*. Animal stocks were screened for microbiological infections monthly.

Fluorescence activated cell sorting (FACS): Pancreata were perfused with 2 ml digestion buffer (1.0 mg/ml collagenase V, 0.2 mg/ml BSA, 0.1 mg/ml soybean trypsin inhibitor, in Hank's buffered saline solution (HBSS)) and the excised organs were incubated at 37°C in a shaking water bath for 30 min. Dissociated cells were filtered through a 40 mm nylon mesh (BD Biosciences, San Jose, CA, USA). Antibodies against GPm6a (1:100, PE conjugated; Bioss Inc, Woburn, MA, USA) and Glut2 (1:100, 647 conjugated; Bioss) were incubated for 60 min to label β-cells. Seven-Aminoactinomycin D (7-AAD) (1:100; BD Biosciences) was added as a viability marker. Cells were sorted at the London Regional Flow Cytometry Facility, Western University, London, ON, Canada cells with a Becton Dickinson FACSAria III cell sorter using FACSDiVa software (v 8.0.1) to separate insulin (GPm6a)-expressing cells co-expressing Glut2 ($Ins^{+}Glut2^{HI}$) from $Ins^{+}Glut2^{LO}$ cells [63].

DNA microarray analysis: Using the RNeasy Plus Mini kit (QIAGEN), total RNA was extracted and purified from $Ins^{+}Glut2^{HI}$ or $Ins^{+}Glut2^{LO}$ fractions that had been separated by FACS. Each RNA sample was hybridized to a GeneChip and processed at the London Regional Genomics Centre, Western University, London, ON. RNA quality was assessed using the Agilent 2100 Bioanalyzer (Agilent Technologies Inc., Palo Alto, CA, USA) and the RNA 6000

Nano kit (Caliper Life Sciences, Mountain View, CA, USA). For expression analysis, the Affymetrix (Santa Clara, CA, USA) GeneChip Mouse Genome 430 2.0 (MOE430 2.0) array was used, which contained 45,000 probe sets representing transcripts and variants from 34,000 mouse genes. All procedures, including cRNA synthesis, labelling, and hybridization to Affymetrix MOE430 2.0 GeneChips were performed as described in the Affymetrix Technical Analysis Manual.

Quantitative polymerase chain reaction (qPCR): RNA was extracted from $Ins^{+}Glut2^{HI}$ and $Ins^{+}Glut2^{LO}$ fractions from 7 day-old neonatal mouse pancreata, isolated islets of Langerhans from pregnant mouse pancreata, and from mouse placentae from control and LP diet-fed animals.

Approximately 3-5 mg of each placenta sample was minced using scissors and placed in RLT lysis buffer containing ß-mercaptoethanol, as per kit instructions (QIAGEN). The tissue was then mechanically homogenized using 18, 20, 21 and 25-gauge needles, sequentially. To further homogenize the resulting lysate was pipetted into a QIAshredder spin column (QIAGEN) and centrifuged for 2 min at 12,500 rpm. The lysate was then transferred to a RNeasy MinElute spin column (QIAGEN) for the extraction of RNA. Finally, the RNA concentration was quantified by spectrophotometry and RNA was stored at -80ºC.

Extracted RNA samples were reverse transcribed to cDNA. Nuclease-free water and supermix provided in an iScript Kit (Bio-Rad, Hercules, CA, USA) were added to each RNA sample (1 µg) and the tubes were then placed in a thermocycler (Eppendorf, Hamburg, Germany) following the kit protocol (5 min at 25ºC, 20 min at 46ºC, and 1 min at 95ºC) to allow for the RT reaction to occur. Resulting cDNA samples were stored at -20ºC. Quantitative PCR experiments were performed on a QuantStudio5 Real-time PCR System (Applied Biosystems,

Waltham, MA, USA). TaqMan primers (assay numbers) for Apelin (Mm00443562_m1), Apela (Mm04278372_m1), Aplnr (Mm00442191_s1), insulin (Mm03038438_m1), TNF-α (Mm00443258_m1), IL-1ß (Mm00434228_m1), IL-6 (Mm00446190_m1) and for the 'housekeeping' control genes, cyclophilin A (cycloA, Mm02342429_g1) and glyceraldehyde-3-phosphate dehydrogenase (GAPDH, Mm99999915_g1) (TaqMan Gene Expression Assays, Applied Biosystems) were used to quantify relative gene expression using the ΔΔ cycle threshold (C_T) method. qPCR was performed using TaqMan Fast Advanced Master Mix (Applied Biosystems) on triplicate samples following manufacturer's instructions. In order to determine which housekeeping gene validated with best precision to the primers, we utilized the average C_T value for each primer at each concentration of cDNA, which was determined by the Quantstudio Design and Analysis Software (Thermo Fisher Scientific, Waltham, MA, USA). ΔC_T values were then calculated by subtracting the C_T of each housekeeping gene from the C_T of the primer of interest at each cDNA concentration. These ΔC_T values were graphed against the log of the cDNA concentration, and the absolute value of the slope had to be <0.1 in order to demonstrate that the efficiencies of target and reference genes were approximately equal.

Immunohistochemistry: Immunofluorescence histochemistry was used to localize Apelin, Aplnr, insulin, glucagon, somatostatin and Glut2 as described [17]. Briefly, background Sniper (Biocare Medical, Concord, CA, USA) was applied to each tissue section for 8 min to reduce non-specific background binding. Antibodies against Apelin (1:100, rabbit polyclonal, Biorbyt, Cambridge, UK), Aplnr (1:100, rabbit polyclonal; Millipore Sigma Canada, Oakville, ON, Canada), insulin (1:50, guinea pig polyclonal, Abcam, Cambridge, UK), glucagon (1:2000, mouse monoclonal; Millipore Sigma Canada), somatostatin (1:100, mouse monoclonal; Santa Cruz Biotechnology, Dallas, TX, USA) or Glut2 (1:200, goat polyclonal; Santa Cruz

Biotechnology) were applied to mouse pancreatic tissues and incubated overnight at 4°C. The following day, secondary antibodies (1:400; Thermo Fisher Scientific) were applied against the primary antibody using 555, 488 and 647 fluorophores, respectively, along with DAPI (4, 6-diamidino-2 phenylindole, dihydrochloride, 1:500; Thermo Fisher Scientific) to counterstain nuclei. Specificity of staining was confirmed by the absence of signal following omission of the secondary antibody for each ligand.

Immunohistochemical staining for Apelin was performed as described above using diaminobenzidine (DAB) as the chromogen. Following prior exposure to 3% (v/v) H_2O_2 to block endogenous peroxidase activity, and then the above primary antibody for apelin, tissues were incubated with biotinylated goat anti-rabbit IgG secondary antibody (1:200; Vector Laboratories, Burlingame, CA, USA) for 2 h at room temperature followed by a 2 h incubation with ExtrAvidin Peroxidase (1:100; Sigma-Aldrich, St. Louis, MO, USA) at room temperature. Apelin was visualized using Liquid DAB Substrate (BioGenex, San Ramon, CA, USA), and tissues were counterstained with Carrazi's hematoxylin. Dehydrated sections were mounted under glass coverslips with Permount (Fisher Scientific, Toronto, ON, Canada).

Islet isolation and tissue culture: Pancreata from neonatal 7-day old mice and pregnant mice were digested with 1 mg/ml collagenase V for 30 min (neonates) or with 0.75 mg/ml for 15 min (adult mice) at 37°C in a shaking water bath. Hank's Balanced Salt Solution (HBSS) containing 5% bovine serum was added to stop digestion and the suspension was passed through a 14-gauge needle to further break up large clumps. Following centrifugation at 1000 rpm for 5 min, islets were separated from acinar tissue using a Dextran density gradient consisting of 27, 23 and 11% concentrations, and were centrifuged for 25 min at 1700 rpm. Islets were collected from the 23/11% interface, washed and hand-picked into a sterile p60 petri dish (Falcon, VWR

International, Mississauga, ON, Canada) containing 5 ml of RPMI 1640 medium containing 10% heat inactivated fetal bovine serum and 6.5 mM glucose. Islets were incubated for 24 h at 37°C and 5% CO_2. The following day, islets were divided into 3 wells of a 6-well ultra-low attachment multi-well plate (Falcon, VWR International) in RPMI medium as above, with and without Pyr-Apelin 13 (100 nM, 1 μM; Sigma), and were and incubated for 48 h.

Following Apelin treatment, islets (approximately 20 islets/treatment) were hand-picked and placed into microfuge tubes containing 1 ml HBSS and centrifuged for 3 min at 1000 rpm. Islets were resuspended in 100 μl HBSS and affixed to glass-bottom dishes (MatTek Life Sciences, Ashland, MA, USA) that were pre-adsorbed with diluted Cell-Tak adhesive (BD Biosciences). Dishes were incubated at room temperature for 30 min to allow the islets to adhere. Islets were then fixed with 4% paraformaldehyde (PFA) for 30 min at room temperature, after which the PFA was removed by washing. The islets were then stored at 4°C in phosphate buffered saline (PBS).

Immunofluorescent staining for insulin and Ki67 was performed on isolated islets to assess the percentage of cells undergoing DNA synthesis. Islets were permeabilized by incubating with 0.3% Triton-X/PBS for 3 h at room temperature, followed by an overnight incubation at 4°C with 5% goat serum/0.15% Triton-X/PBS to block non-specific staining. The next day primary antibodies to insulin (1:200, rabbit polyclonal; Santa Cruz Biotech) and Ki67 (1:100, rat monoclonal; Agilent/Dako) diluted in 0.2% Triton-X/1% BSA were added and incubated overnight at 4°C. Excess antibodies were removed by washing and islets were incubated overnight at 4°C with secondary antibodies (488 and 647 fluorophores, 1:400; Thermo Fisher Scientific). The following day, DAPI (1:500; Thermo Fisher Scientific) was added for 10 min to counterstain nuclei.

MTT (4, 6-diamidino-2 phenylindole, dihydrochloride) assay: INS1E cells were plated at a concentration of 2×10^3 cells/well in 100 µl culture medium (RPMI 1640 supplemented with 10 mM Hepes, 10% fetal bovine serum, 11.1 mM glucose, 2 mM l-glutamine, 1 mM sodium pyruvate and 50 µM beta-mercaptoethanol) containing Apelin (10 nM) or Apela (4 nM) with or without the specific Aplnr antagonist, ML221 (10 µM; BioTechne, Minneapolis, MN, USA) and were incubated at 37°C and 5% CO_2 for 48h in microplates (tissue culture grade, 96 wells, flat bottom) using the manufacturer's protocol (Millipore-Sigma). Spectrophotometric absorbance of the purple formazan crystal product was measured using a microplate reader at an absorbance wavelength of 590 nm.
