# Supplementary material for: Ontology of the apelinergic system in mouse pancreas during pregnancy and relationship with β-cell mass
Source: Sci Rep. 2021 Jul 29;11:15475. doi: 10.1038/s41598-021-94725-0 (PMC8322410; doi:10.1038/s41598-021-94725-0)
Supplement: Supplementary file 4 — Supplementary Table 1. [file 41598_2021_94725_MOESM4_ESM.pdf]

Column#	Transcript ID	Gene_assignment	Gene Symbol	RefSeq	Fold-Difference(GLUT2^{LO} vs. GLUT2^{HI})
27433	17428883	---		---	97
35735	17510338	NM_032398 // Plvap // plasmalemma vesicle associated protein // 8 B3.3|8 34.43 cM // 84	Plvap	NM_032398	67
21845	17372725	NM_011784 // Aplnr // apelin receptor // 2|2 E1 // 23796 /// ENSMUST00000057019 // Apln	Aplnr	NM_011784	48
9784	17256697	NM_009675 // Aoc3 // amine oxidase, copper containing 3 // 11|11 B2-B5 // 11754 /// XM_	Aoc3	NM_009675	47
7862	17237451	NM_029928 // Ptprb // protein tyrosine phosphatase, receptor type, B // 10|10 D2 // 192	Ptprb	NM_029928	43
3027	17206137	---		---	41
16972	17325324	NM_173869 // Stfa2l1 // stefin A2 like 1 // 16|16 B3 // 268885 /// ENSMUST00000079184 /	Stfa2l1	NM_173869	41
26116	17415469	NM_001290549 // Tek // endothelial-specific receptor tyrosine kinase // 4 C5|4 43.34 cM	Tek	NM_001290549	41
24122	17395928	NM_009236 // Sox18 // SRY (sex determining region Y)-box 18 // 2 H4|2 103.71 cM // 2067	Sox18	NM_009236	39
11491	17274703	NM_181395 // Pxdn // peroxidasin homolog (Drosophila) // 12|12 A2 // 69675 /// XM_00651	Pxdn	NM_181395	38
12712	17285225	XM_011244360 // LOC105245453 // nidogen-1-like // --- // 105245453 /// XM_011244361 //	LOC105245453	XM_011244360	36
3028	17206139	---		---	36
18271	17337770	NM_001081178 // Adgrf5 // adhesion G protein-coupled receptor F5 // 17|17 B3 // 224792	Adgrf5	NM_001081178	35
23581	17390879	NM_007993 // Fbn1 // fibrillin 1 // 2 F|2 61.38 cM // 14118 /// XM_006498747 // Fbn1 //	Fbn1	NM_007993	35
33423	17488179	NM_001113549 // Ltbp4 // latent transforming growth factor beta binding protein 4 // 7|	Ltbp4	NM_001113549	34
24820	17402981	NM_001163522 // Emcn // endomucin // 3|3 G3 // 59308 /// NM_016885 // Emcn // endomucin	Emcn	NM_001163522	34
29278	17448924	NM_010612 // Kdr // kinase insert domain protein receptor // 5 C3.3|5 40.23 cM // 16542	Kdr	NM_010612	33
29716	17453454	NM_007925 // Eln // elastin // 5 G2|5 74.76 cM // 13717 /// XM_006504360 // Eln // elas	Eln	NM_007925	33
27431	17428881	---		---	32
34608	17498962	NM_009932 // Col4a2 // collagen, type IV, alpha 2 // 8 A1.1|8 5.62 cM // 12827 /// ENSM	Col4a2	NM_009932	29
30999	17465620	NM_013723 // Podxl // podocalyxin-like // 6 A3.3|6 12.57 cM // 27205 /// ENSMUST0000002	Podxl	NM_013723	28
12663	17284660	AF045497 // Ighm // immunoglobulin heavy constant mu // 12 62.1 cM|12 F1-2 // 16019 ///	Ighm	AF045497	28
6276	17221014	NM_001111059 // Cd34 // CD34 antigen // 1 H6|1 98.38 cM // 12490 /// NM_133654 // Cd34	Cd34	NM_001111059	28
18485	17340197	NM_010137 // Epas1 // endothelial PAS domain protein 1 // 17|17 E4 // 13819 /// ENSMUST	Epas1	NM_010137	28
11184	17270937	NM_001032378 // Pecam1 // platelet/endothelial cell adhesion molecule 1 // 11|11 E1 //	Pecam1	NM_001032378	28
33236	17486807	NM_153068 // Ehd2 // EH-domain containing 2 // 7 A2|7 8.65 cM // 259300 /// XM_01125058	Ehd2	NM_153068	28
12608	17284512	OTTMUST00000130801 // Ighv1-9 // immunoglobulin heavy variable V1-9 // 12|12 F2 // 6684	Ighv1-9	OTTMUST0000013<br>0801	27
6033	17218927	NM_019759 // Dpt // dermatopontin // 1|1 H2 // 56429 /// ENSMUST00000027861 // Dpt // d	Dpt	NM_019759	27
22230	17376549	NM_001126338 // Prnd // prion protein dublet // 2 F2|2 64.07 cM // 26434 /// NM_0012782	Prnd	NM_001126338	27
26178	17416234	NM_080555 // Plpp3 // phospholipid phosphatase 3 // 4 C6|4 49.18 cM // 67916 /// ENSMUS	Plpp3	NM_080555	26
23189	17387517	NM_009776 // Serping1 // serine (or cysteine) peptidase inhibitor, clade G, member 1 //	Serping1	NM_009776	26
26563	17420171	NM_008305 // Hspg2 // perlecan (heparan sulfate proteoglycan 2) // 4 D3|4 69.93 cM // 1	Hspg2	NM_008305	26
16295	17318100	NM_001099217 // Ly6c2 // lymphocyte antigen 6 complex, locus C2 // 15|15 D3 // 10004154	Ly6c2	NM_001099217	26
27448	17429206	NM_011587 // Tie1 // tyrosine kinase with immunoglobulin-like and EGF-like domains 1 //	Tie1	NM_011587	26
35429	17507377	NM_009931 // Col4a1 // collagen, type IV, alpha 1 // 8 A1.1|8 5.53 cM // 12826 /// XR_3	Col4a1	NM_009931	25
32598	17480485	NM_001113379 // Lrrc32 // leucine rich repeat containing 32 // 7 E2|7 53.86 cM // 43421	Lrrc32	NM_001113379	25

38289	17536067	NM_138751 // Tmem47 // transmembrane protein 47 // X|X A7.2 // 192216 /// ENSMUST000000	Tmem47	NM_138751	25
7030	17229481	NM_022563 // Ddr2 // discoidin domain receptor family, member 2 // 1 76.84 cM|1 H1-H5 /	Ddr2	NM_022563	24
24874	17403605	NM_133222 // Adgrl4 // adhesion G protein-coupled receptor L4 // 3|3 H3-H4 // 170757 //	Adgrl4	NM_133222	24
24926	17404091	NM_024406 // Fabp4 // fatty acid binding protein 4, adipocyte // 3 A1|3 2.56 cM // 1177	Fabp4	NM_024406	24
30022	17456934	NM_001252292 // Mest // mesoderm specific transcript // 6 12.53 cM|6 B1 // 17294 /// NM	Mest	NM_001252292	23
12217	17281350	NM_025809 // Clec14a // C-type lectin domain family 14, member a // 12|12 C1 // 66864 /	Clec14a	NM_025809	23
6051	17219139	NM_001313705 // Rgs5 // regulator of G-protein signaling 5 // 1 76.84 cM|1 H2 // 19737	Rgs5	NM_001313705	23
12964	17287160	NM_001172481 // Aspn // asporin // 13|13 B1 // 66695 /// NM_025711 // Aspn // asporin /	Aspn	NM_001172481	23
30251	17458813	NM_007472 // Aqp1 // aquaporin 1 // 6 B3|6 27.38 cM // 11826 /// ENSMUST00000004774 //	Aqp1	NM_007472	22
3013	17206107	---		---	22
35163	17504399	NM_009868 // Cdh5 // cadherin 5 // 8 D3|8 53.04 cM // 12562 /// XM_006530630 // Cdh5 //	Cdh5	NM_009868	22
29461	17450387	NM_010097 // Sparcl1 // SPARC-like 1 // 5 50.55 cM|5 E4 // 13602 /// XM_006534768 // Sp	Sparcl1	NM_010097	22
25930	17413866	NM_009928 // Col15a1 // collagen, type XV, alpha 1 // 4|4 B1-B3 // 12819 /// XM_0065376	Col15a1	NM_009928	22
5499	17212719	NM_138741 // Sdpr // serum deprivation response // 1|1 C1.1 // 20324 /// ENSMUST0000005	Sdpr	NM_138741	21
5488	17212461	NM_009930 // Col3a1 // collagen, type III, alpha 1 // 1 C1.1|1 23.67 cM // 12825 /// EN	Col3a1	NM_009930	21
9647	17255260	NM_007742 // Col1a1 // collagen, type I, alpha 1 // 11 59.01 cM|11 D // 12842 /// ENSMU	Col1a1	NM_007742	21
11499	17274775	NM_008482 // Lamb1 // laminin B1 // 12 A2-A3|12 13.39 cM // 16777 /// XM_006514992 // L	Lamb1	NM_008482	21
20811	17362595	NM_019699 // Fads2 // fatty acid desaturase 2 // 19|19 B // 56473 /// ENSMUST0000002556	Fads2	NM_019699	21
17810	17333318	---		---	21
16954	17325069	NM_175256 // Heg1 // HEG homolog 1 (zebrafish) // 16|16 B3 // 77446 /// XM_006522715 //	Heg1	NM_175256	21
29863	17455346	NM_010228 // Flt1 // FMS-like tyrosine kinase 1 // 5 87.01 cM|5 G // 14254 /// XM_00650	Flt1	NM_010228	21
27582	17430576	NM_001168333 // Tinagl1 // tubulointerstitial nephritis antigen-like 1 // 4|4 D3 // 942	Tinagl1	NM_001168333	21
31206	17467384	OTTMUST00000132012 // Igkv10-96 // immunoglobulin kappa variable 10-96 // 6|6 C1 // 692	Igkv10-96	OTTMUST0000013<br>2012	21
11897	17278775	NR_028433 // AF357355 // snoRNA AF357355 // 12|12 F1 // 100303646 /// AF357355 // AF357	AF357355	NR_028433	21
7731	17236182	NM_011595 // Timp3 // tissue inhibitor of metalloproteinase 3 // 10 42.83 cM|10 C1-D1 /	Timp3	NM_011595	20
26278	17417407	NM_181585 // Pik3r3 // phosphatidylinositol 3 kinase, regulatory subunit, polypeptide 3	Pik3r3	NM_181585	20
33836	17491853	NM_001163574 // Tjp1 // tight junction protein 1 // 7 C|7 35.02 cM // 21872 /// NM_0093	Tjp1	NM_001163574	20
7354	17232593	NM_010681 // Lama4 // laminin, alpha 4 // 10 B1|10 20.02 cM // 16775 /// ENSMUST0000001	Lama4	NM_010681	20
30628	17462145	NM_001166580 // 8430408G22Rik // RIKEN cDNA 8430408G22 gene // 6|6 E3 // 213393 /// NM_	8430408G22Rik	NM_001166580	20
21966	17373550	NM_001025246 // Trp53i11 // transformation related protein 53 inducible protein 11 // 2	Trp53i11	NM_001025246	20
8934	17247948	NM_146015 // Efemp1 // epidermal growth factor-containing fibulin-like extracellular ma	Efemp1	NM_146015	20
30304	17459291	M20830 // Igkv1-135 // immunoglobulin kappa variable 1-135 // 6 C1|6 // 243420 /// OTTM	Igkv1-135	M20830	20
17135	17326801	NM_023844 // Jam2 // junction adhesion molecule 2 // 16|16 C3.3 // 67374 /// XM_0065230	Jam2	NM_023844	20
8162	17239817	NM_134005 // Enpp3 // ectonucleotide pyrophosphatase/phosphodiesterase 3 // 10|10 A4 //	Enpp3	NM_134005	20
35420	17507321	NM_010111 // Efnb2 // ephrin B2 // 8 A1.1|8 3.42 cM // 13642 /// XM_006508694 // Efnb2	Efnb2	NM_010111	20
27190	17426765	NM_001113209 // Nfib // nuclear factor I/B // 4 38.4 cM|4 C4-C6 // 18028 /// NM_0011132	Nfib	NM_001113209	19
29959	17456161	NM_001243064 // Cav1 // caveolin 1, caveolae protein // 6|6 A2 // 12389 /// NM_007616 /	Cav1	NM_001243064	19
6589	17224180	NM_010518 // Igfbp5 // insulin-like growth factor binding protein 5 // 1 C3|1 36.94 cM	Igfbp5	NM_010518	19
29926	17455801	NM_007743 // Col1a2 // collagen, type I, alpha 2 // 6 A1|6 1.81 cM // 12843 /// ENSMUST	Col1a2	NM_007743	19
37433	17527661	NM_001195431 // Islr // immunoglobulin superfamily containing leucine-rich repeat // 9|	Islr	NM_001195431	18

36939	17522369	NM_008694 // Ngp // neutrophilic granule protein // 9|9 F2 // 18054 /// ENSMUST00000035	Ngp	NM_008694	18
18272	17337794	ENSMUST00000082648 // Gm25135 // predicted gene, 25135 [Source:MGI Symbol;Acc:MGI:54549	Gm25135	ENSMUST0000008 2648	18
11888	17278757	ENSMUST00000082518 // Gm24564 // predicted gene, 24564 [Source:MGI Symbol;Acc:MGI:54543	Gm24564	ENSMUST0000008 2518	18
32560	17480036	NM_008055 // Fzd4 // frizzled homolog 4 (Drosophila) // 7 E1|7 49.32 cM // 14366 /// XM	Fzd4	NM_008055	18
28659	17442834	NM_001081342 // Adgrd1 // adhesion G protein-coupled receptor D1 // 5|5 G1.3 // 243277	Adgrd1	NM_001081342	18
31442	17469136	NM_175314 // Adamts9 // a disintegrin-like and metallopeptidase (reprolysin type) with	Adamts9	NM_175314	17
36429	17516518	NM_023061 // Mcam // melanoma cell adhesion molecule // 9|9 A5.1 // 84004 /// XM_006510	Mcam	NM_023061	17
6984	17229036	NM_018881 // Fmo2 // flavin containing monooxygenase 2 // 1|1 H1 // 55990 /// XM_006496	Fmo2	NM_018881	17
12003	17279499	NM_024223 // Crip2 // cysteine rich protein 2 // 12 F1|12 61.57 cM // 68337 /// XM_0065	Crip2	NM_024223	17
7793	17236811	NM_008524 // Lum // lumican // 10 C3|10 50.32 cM // 17022 /// ENSMUST00000038160 // Lum	Lum	NM_008524	17
11890	17278761	NR_046302 // AF357425 // snoRNA AF357425 // 12 60.5 cM // 449562	AF357425	NR_046302	17
30524	17460891	NM_001081437 // Fbln2 // fibulin 2 // 6 D|6 40.42 cM // 14115 /// NM_007992 // Fbln2 //	Fbln2	NM_001081437	17
8067	17238934	XM_006512458 // Syne1 // spectrin repeat containing, nuclear envelope 1 // 10|10 A1 //	Syne1	XM_006512458	17
13985	17296388	NM_001033228 // Itga1 // integrin alpha 1 // 13 D2.2|13 64.61 cM // 109700 /// ENSMUST0	Itga1	NM_001033228	16
24493	17399823	NM_013650 // S100a8 // S100 calcium binding protein A8 (calgranulin A) // 3 39.9 cM|3 F	S100a8	NM_013650	16
8060	17238920	XM_006512458 // Syne1 // spectrin repeat containing, nuclear envelope 1 // 10|10 A1 //	Syne1	XM_006512458	16
28726	17443539	NM_001159571 // Ephb4 // Eph receptor B4 // 5|5 G2 // 13846 /// NM_010144 // Ephb4 // E	Ephb4	NM_001159571	16
12589	17284460	OTTMUST00000130540 // Ighv9-3 // immunoglobulin heavy variable V9-3 // 12|12 F2 // 7808	Ighv9-3	OTTMUST0000013 0540	16
36326	17516047	NM_001309390 // Robo4 // roundabout homolog 4 (Drosophila) // 9|9 A4 // 74144 /// NM_02	Robo4	NM_001309390	16
38238	17535558	NM_007542 // Bgn // biglycan // X 37.33 cM|X B // 12111 /// XM_006527758 // Bgn // bigl	Bgn	NM_007542	16
7792	17236800	NM_001190451 // Dcn // decorin // 10 C3|10 50.27 cM // 13179 /// NM_007833 // Dcn // de	Dcn	NM_001190451	16
12602	17284498	OTTMUST00000130737 // Ighv10-1 // immunoglobulin heavy variable 10-1 // 12|12 F2 // 380	Ighv10-1	OTTMUST0000013 0737	16
19310	17348138	NM_001081963 // 9430020K01Rik // RIKEN cDNA 9430020K01 gene // 18|18 A1 // 240185 /// X	9430020K01Rik	NM_001081963	16
38959	17541681	NM_016697 // Gpc3 // glypican 3 // X|X A3.3 // 14734 /// XM_006541429 // Gpc3 // glypic	Gpc3	NM_016697	15
38625	17538803	---		---	15
5362	17211131	NM_001033636 // Prex2 // phosphatidylinositol-3,4,5-trisphosphate-dependent Rac exchang	Prex2	NM_001033636	15
24288	17397575	NM_001198765 // Postn // periostin, osteoblast specific factor // 3|3 C // 50706 /// NM	Postn	NM_001198765	15
17517	17330625	NM_010818 // Cd200 // CD200 antigen // 16 29.53 cM|16 A1 // 17470 /// XR_384531 // Cd20	Cd200	NM_010818	15
23378	17388435	NM_183180 // Tspan18 // tetraspanin 18 // 2|2 E1 // 241556 /// XM_006499474 // Tspan18	Tspan18	NM_183180	15
25163	17406221	NM_021896 // Gucy1a3 // guanylate cyclase 1, soluble, alpha 3 // 3|3 E3 // 60596 /// XM	Gucy1a3	NM_021896	15
30669	17462663	NM_015776 // Mfap5 // microfibrillar associated protein 5 // 6|6 F1 // 50530 /// XM_006	Mfap5	NM_015776	15
36597	17518310	ENSMUST00000083818 // Gm23344 // predicted gene, 23344 [Source:MGI Symbol;Acc:MGI:54531	Gm23344	ENSMUST0000008 3818	15
11882	17278715	NR_028261 // Rian // RNA imprinted and accumulated in nucleus // 12 F1|12 60.41 cM // 7	Rian	NR_028261	15
12966	17287175	NM_008760 // Ogn // osteoglycin // 13|13 A5 // 18295 /// ENSMUST00000021822 // Ogn // o	Ogn	NM_008760	15
23669	17391486	---		---	15
9871	17257444	NM_001281819 // Ace // angiotensin I converting enzyme (peptidyl-dipeptidase A) 1 // 11	Ace	NM_001281819	15
22078	17374488	NM_001313914 // Thbs1 // thrombospondin 1 // 2 59.34 cM|2 F1-F3 // 21825 /// NM_011580	Thbs1	NM_001313914	15

9029	17248691	NM_001290709 // Ebf1 // early B cell factor 1 // 11 B1.1|11 26.45 cM // 13591 /// NM_00	Ebf1	NM_001290709	15
16992	17325514	NM_008047 // Fstl1 // follistatin-like 1 // 16 B3|16 26.48 cM // 14314 /// ENSMUST00000	Fstl1	NM_008047	15
12716	17285244	NM_010917 // Nid1 // nidogen 1 // 13 A1|13 5.26 cM // 18073 /// ENSMUST00000005532 // N	Nid1	NM_010917	14
23629	17391440	---		---	14
12550	17284354	AF099087 // Igh-VX24 // immunoglobulin heavy chain (X24 family) // 12 F2|12 // 195176 /	Igh-VX24	AF099087	14
23168	17387316	NM_001290745 // Nckap1 // NCK-associated protein 1 // 2 C3|2 48.21 cM // 50884 /// NM_0	Nckap1	NM_001290745	14
36665	17519184	NM_010890 // Nedd4 // neural precursor cell expressed, developmentally down-regulated 4	Nedd4	NM_010890	14
30437	17460185	NM_001077694 // Dysf // dysferlin // 6 C3|6 36.14 cM // 26903 /// NM_001310152 // Dysf	Dysf	NM_001077694	14
15911	17314190	NM_021423 // Shank3 // SH3/ankyrin domain gene 3 // 15|15 E3 // 58234 /// XM_006521220	Shank3	NM_021423	14
27149	17426237	NM_007443 // Ambp // alpha 1 microglobulin/bikunin // 4 33.96 cM|4 C1-C3 // 11699 /// E	Ambp	NM_007443	14
19609	17351027	NM_001146268 // Pdgfrb // platelet derived growth factor receptor, beta polypeptide //	Pdgfrb	NM_001146268	14
22248	17376685	NM_001145830 // Plcb1 // phospholipase C, beta 1 // 2 F3|2 65.66 cM // 18795 /// NM_019	Plcb1	NM_001145830	14
35114	17503825	NM_008610 // Mmp2 // matrix metallopeptidase 2 // 8 C5|8 44.99 cM // 17390 /// XM_00653	Mmp2	NM_008610	14
8061	17238922	XM_006512458 // Syne1 // spectrin repeat containing, nuclear envelope 1 // 10|10 A1 //	Syne1	XM_006512458	14
28422	17439830	NM_001204201 // Spp1 // secreted phosphoprotein 1 // 5 E5|5 50.68 cM // 20750 /// NM_00	Spp1	NM_001204201	14
14711	17302289	NM_001013753 // Pcdh17 // protocadherin 17 // 14|14 D3 // 219228 /// XM_006518905 // Pc	Pcdh17	NM_001013753	14
29670	17452957	NM_001205082 // Scarb1 // scavenger receptor class B, member 1 // 5 64.11 cM|5 G1.1 //	Scarb1	NM_001205082	14
3022	17206127	---		---	14
38934	17541378	NM_013912 // Apln // apelin // X|X A3.2 // 30878 /// ENSMUST00000039026 // Apln // apel	Apln	NM_013912	14
7226	17231477	NM_026793 // Myct1 // myc target 1 // 10|10 A1 // 68632 /// ENSMUST00000051809 // Myct1	Myct1	NM_026793	14
6077	17219362	NM_172647 // F11r // F11 receptor // 1 H2|1 79.43 cM // 16456 /// ENSMUST00000043839 //	F11r	NM_172647	14
10161	17260474	NM_008343 // Igfbp3 // insulin-like growth factor binding protein 3 // 11 A1|11 4.75 cM	Igfbp3	NM_008343	14
29216	17448756	---		---	14
15782	17312774	NM_027219 // Cdc42ep1 // CDC42 effector protein (Rho GTPase binding) 1 // 15|15 E1 // 1	Cdc42ep1	NM_027219	13
7436	17233347	NM_010288 // Gja1 // gap junction protein, alpha 1 // 10 B4|10 28.64 cM // 14609 /// XM	Gja1	NM_010288	13
12585	17284450	M19571 // Igh-VJ558 // immunoglobulin heavy chain (J558 family) // 12 F2|12 // 16061 //	Igh-VJ558	M19571	13
30320	17459338	AF154883 // Igk-V1 // immunoglobulin kappa chain variable 1 (V1) // --- // 16081 /// AF	Igk-V1	AF154883	13
16066	17315743	NM_001310469 // Osmr // oncostatin M receptor // 15 A1|15 3.3 cM // 18414 /// NM_011019	Osmr	NM_001310469	13
18542	17340673	NM_001081416 // Fndc1 // fibronectin type III domain containing 1 // 17|17 A1 // 68655	Fndc1	NM_001081416	13
30629	17462149	NM_001012477 // Cxcl12 // chemokine (C-X-C motif) ligand 12 // 6 54.81 cM|6 F1 // 20315	Cxcl12	NM_001012477	13
34558	17498467	NM_001242349 // Ano1 // anoctamin 1, calcium activated chloride channel // 7|7 F5 // 10	Ano1	NM_001242349	13
15618	17311179	NM_001162494 // Fzd6 // frizzled homolog 6 (Drosophila) // 15 B3.1|15 15.22 cM // 14368	Fzd6	NM_001162494	13
16293	17318083	NM_001271416 // Ly6a // lymphocyte antigen 6 complex, locus A // 15 D3|15 34.29 cM // 1	Ly6a	NM_001271416	13
14195	17298825	NM_153127 // Mmrn2 // multimerin 2 // 14|14 B // 105450 /// ENSMUST00000111908 // Mmrn2	Mmrn2	NM_153127	13
35143	17504190	NM_001198894 // Adgrg1 // adhesion G protein-coupled receptor G1 // 8 D1|8 47.12 cM //	Adgrg1	NM_001198894	13
36831	17520905	NM_033314 // Slco2a1 // solute carrier organic anion transporter family, member 2a1 //	Slco2a1	NM_033314	13
30315	17459324	OTTMUST00000131864 // Igkv1-117 // immunoglobulin kappa variable 1-117 // 6 C1|6 31.07	Igkv1-117	OTTMUST0000013<br>1864	13
12660	17284652	OTTMUST00000131426 // Ighv1-78 // immunoglobulin heavy variable 1-78 // 12|12 F2 // 213	Ighv1-78	OTTMUST0000013<br>1426	13
17649	17331692	NM_144853 // Cyyr1 // cysteine and tyrosine-rich protein 1 // 16 C3.3|16 47.45 cM // 22	Cyyr1	NM_144853	13

17651	17331705	NM_009621 // Adamts1 // a disintegrin-like and metallopeptidase (reprolysin type) with	Adamts1	NM_009621	13
33143	17486099	NM_011769 // Zim1 // zinc finger, imprinted 1 // 7 A1|7 3.87 cM // 22776 /// XM_0112504	Zim1	NM_011769	13
12624	17284557	OTTMUST00000130897 // Ighv1-39 // immunoglobulin heavy variable 1-39 // 12|12 F2 // 780	Ighv1-39	OTTMUST0000013 0897	13
28243	17438246	NM_001083316 // Pdgfra // platelet derived growth factor receptor, alpha polypeptide //	Pdgfra	NM_001083316	12
3014	17206109	---		---	12
24241	17397185	NM_183221 // Fat4 // FAT tumor suppressor homolog 4 (Drosophila) // 3|3 B // 329628 ///	Fat4	NM_183221	12
39225	17544078	NM_008409 // Itm2a // integral membrane protein 2A // X|X A2-A3 // 16431 /// ENSMUST000	Itm2a	NM_008409	12
16007	17315272	NM_153533 // Tns2 // tensin 2 // 15 F3|15 57.29 cM // 209039 /// XM_006520691 // Tns2 /	Tns2	NM_153533	12
30334	17459377	OTTMUST00000133028 // Igkv6-25 // immunoglobulin kappa chain variable 6-25 // 6|6 C1 //	Igkv6-25	OTTMUST0000013 3028	12
16976	17325347	NM_001082543 // Stfa1 // stefin A1 // 16 B3|16 25.45 cM // 20861 /// ENSMUST00000042097	Stfa1	NM_001082543	12
29169	17448415	NM_001201413 // Apbb2 // amyloid beta (A4) precursor protein-binding, family B, member	Apbb2	NM_001201413	12
16535	17320947	NM_027052 // Slc38a4 // solute carrier family 38, member 4 // 15|15 F1 // 69354 /// XM_	Slc38a4	NM_027052	12
17070	17326318	NM_001014399 // Abi3bp // ABI gene family, member 3 (NESH) binding protein // 16|16 C1.	Abi3bp	NM_001014399	12
15495	17309580	NM_001081039 // Dock9 // dedicator of cytokinesis 9 // 14 E5|14 65.28 cM // 105445 ///	Dock9	NM_001081039	12
38827	17540436	NM_175228 // 4930578C19Rik // RIKEN cDNA 4930578C19 gene // X|X A1.3 // 75905 /// NR_13	4930578C19Rik	NM_175228	12
28711	17443380	NM_175309 // Upk3b // uroplakin 3B // 5|5 G2 // 100647 /// ENSMUST00000062606 // Upk3b	Upk3b	NM_175309	12
25060	17405355	NM_008536 // Tm4sf1 // transmembrane 4 superfamily member 1 // 3|3 D // 17112 /// XM_00	Tm4sf1	NM_008536	12
22204	17376270	NR_028547 // Snord110 // small nucleolar RNA, C/D box 110 // 2|2 // 100217452	Snord110	NR_028547	12
14508	17300279	NM_008608 // Mmp14 // matrix metallopeptidase 14 (membrane-inserted) // 14 C2|14 27.79	Mmp14	NM_008608	12
31379	17468573	NM_054041 // Antxr1 // anthrax toxin receptor 1 // 6|6 D1 // 69538 /// XM_006506564 //	Antxr1	NM_054041	12
16773	17323192	NM_011415 // Snai2 // snail family zinc finger 2 // 16 A1|16 10.07 cM // 20583 /// ENSM	Snai2	NM_011415	12
17738	17332358	NM_001302152 // Erg // avian erythroblastosis virus E-26 (v-ets) oncogene related // 16	Erg	NM_001302152	12
8846	17247039	NM_001291857 // Aebp1 // AE binding protein 1 // 11|11 A1 // 11568 /// NM_009636 // Aeb	Aebp1	NM_001291857	12
6487	17222837	NM_001161817 // Myo1b // myosin IB // 1 C1.1|1 26.58 cM // 17912 /// NM_001290982 // My	Myo1b	NM_001161817	12
35777	17510685	NM_010332 // Ednra // endothelin receptor type A // 8|8 C2 // 13617 /// ENSMUST00000034	Ednra	NM_010332	12
20971	17364098	NM_007392 // Acta2 // actin, alpha 2, smooth muscle, aorta // 19|19 C3 // 11475 /// XM_	Acta2	NM_007392	12
24184	17396383	NM_009425 // Tnfsf10 // tumor necrosis factor (ligand) superfamily, member 10 // 3|3 A3	Tnfsf10	NM_009425	12
6273	17220974	NM_008882 // Plxna2 // plexin A2 // 1|1 H6 // 18845 /// XM_006497240 // Plxna2 // plexi	Plxna2	NM_008882	12
13221	17289432	NM_001004149 // Zfp366 // zinc finger protein 366 // 13|13 D1 // 238803 /// ENSMUST0000	Zfp366	NM_001004149	11
39346	17545106	NM_007603 // Capn6 // calpain 6 // X|X F2 // 12338 /// ENSMUST00000087316 // Capn6 // c	Capn6	NM_007603	11
34014	17493658	NM_001111043 // Serpinh1 // serine (or cysteine) peptidase inhibitor, clade H, member 1	Serpinh1	NM_001111043	11
15203	17306877	NM_080435 // Adcy4 // adenylate cyclase 4 // 14|14 D3 // 104110 /// XM_006518323 // Adc	Adcy4	NM_080435	11
24750	17402193	NM_172525 // Arhgap29 // Rho GTPase activating protein 29 // 3|3 G1 // 214137 /// XM_00	Arhgap29	NM_172525	11
9799	17256870	NM_001160711 // Cd300lg // CD300 antigen like family member G // 11 D|11 65.48 cM // 52	Cd300lg	NM_001160711	11
37151	17524775	NM_177030 // Dock6 // dedicator of cytokinesis 6 // 9|9 A3 // 319899 /// XM_006510422 /	Dock6	NM_177030	11
25426	17408483	NM_011197 // Ptgfrn // prostaglandin F2 receptor negative regulator // 3|3 F3 // 19221	Ptgfrn	NM_011197	11
30499	17460665	NM_001166249 // Mgll // monoglyceride lipase // 6|6 D1 // 23945 /// NM_001166250 // Mgl	Mgll	NM_001166249	11
40645	17549342	---		---	11
31290	17467806	NM_001079822 // Tcf7l1 // transcription factor 7 like 1 (T cell specific, HMG box) // 6	Tcf7l1	NM_001079822	11

21206	17366670	NM_172471 // Itih5 // inter-alpha (globulin) inhibitor H5 // 2|2 A1 // 209378 /// XM_00	Itih5	NM_172471	11
41084	17550446	---		---	11
33630	17490274	NM_019866 // Spib // Spi-B transcription factor (Spi-1/PU.1 related) // 7 B4|7 28.83 cM	Spib	NM_019866	11
37968	17533269	NM_019634 // Tspan7 // tetraspanin 7 // X|X A1.3-A2 // 21912 /// XM_006527583 // Tspan7	Tspan7	NM_019634	11
29468	17450461	NM_001256005 // Gbp4 // guanylate binding protein 4 // 5 E5|5 50.68 cM // 17472 /// NM_	Gbp4	NM_001256005	11
38401	17537112	NM_175271 // Lpar4 // lysophosphatidic acid receptor 4 // X|X D // 78134 /// ENSMUST000	Lpar4	NM_175271	11
32381	17477930	NM_028544 // Rasip1 // Ras interacting protein 1 // 7|7 B2 // 69903 /// ENSMUST00000057	Rasip1	NM_028544	11
16315	17318312	NM_144847 // Nrbp2 // nuclear receptor binding protein 2 // 15|15 D3 // 223649 /// XM_0	Nrbp2	NM_144847	11
34634	17499335	NM_001143671 // Tmem255b // transmembrane protein 255B // 8|8 A1.1 // 272465 /// NM_001	Tmem255b	NM_001143671	11
36719	17519821	NM_001039546 // Myo6 // myosin VI // 9 E1|9 43.98 cM // 17920 /// XM_006510835 // Myo6	Myo6	NM_001039546	11
10695	17266038	NM_029658 // Fam101b // family with sequence similarity 101, member B // 11|11 B4 // 76	Fam101b	NM_029658	11
8391	17242177	NM_009933 // Col6a1 // collagen, type VI, alpha 1 // 10 B5-C1|10 39.71 cM // 12833 ///	Col6a1	NM_009933	11
9076	17249036	NM_013529 // Gfpt2 // glutamine fructose-6-phosphate transaminase 2 // 11 B1.2|11 29.9	Gfpt2	NM_013529	11
38191	17535171	ENSMUST00000122510 // Gm24598 // predicted gene, 24598 [Source:MGI Symbol;Acc:MGI:54543	Gm24598	ENSMUST0000012 2510	11
26566	17420347	NM_199307 // Ece1 // endothelin converting enzyme 1 // 4|4 D3 // 230857 /// XM_00653876	Ece1	NM_199307	11
2852	17205781	---		---	11
15660	17311551	NM_181277 // Col14a1 // collagen, type XIV, alpha 1 // 15|15 D // 12818 /// XM_00652038	Col14a1	NM_181277	11
12150	17280749	NM_025359 // Tspan13 // tetraspanin 13 // 12|12 B2 // 66109 /// ENSMUST00000020896 // T	Tspan13	NM_025359	11
29290	17449084	NM_001159518 // Igfbp7 // insulin-like growth factor binding protein 7 // 5|5 C3.3 // 2	Igfbp7	NM_001159518	11
15657	17311519	NM_010930 // Nov // nephroblastoma overexpressed gene // 15 D1|15 21.49 cM // 18133 ///	Nov	NM_010930	11
26048	17414984	NM_026821 // Lurap1l // leucine rich adaptor protein 1-like // 4 C3|4 37.91 cM // 52829	Lurap1l	NM_026821	11
12614	17284530	OTTMUST00000130843 // Ighv1-19 // immunoglobulin heavy variable V1-19 // 12|12 F2 // 38	Ighv1-19	OTTMUST0000013 0843	11
13938	17295817	NM_175171 // Mast4 // microtubule associated serine/threonine kinase family member 4 //	Mast4	NM_175171	11
13503	17291525	NM_009238 // Sox4 // SRY (sex determining region Y)-box 4 // 13 12.87 cM|13 A3-A5 // 20	Sox4	NM_009238	11
8065	17238930	XM_006512458 // Syne1 // spectrin repeat containing, nuclear envelope 1 // 10|10 A1 //	Syne1	XM_006512458	11
36329	17516087	NM_020518 // Vsig2 // V-set and immunoglobulin domain containing 2 // 9|9 B // 57276 //	Vsig2	NM_020518	11
33144	17486110	NM_008817 // Peg3 // paternally expressed 3 // 7 3.89 cM|7 A2-B1 // 18616 /// ENSMUST00	Peg3	NM_008817	10
17652	17331720	NM_011782 // Adamts5 // a disintegrin-like and metallopeptidase (reprolysin type) with	Adamts5	NM_011782	10
36328	17516074	NM_027102 // Esam // endothelial cell-specific adhesion molecule // 9|9 B // 69524 ///	Esam	NM_027102	10
12575	17284423	OTTMUST00000130433 // Ighv5-17 // immunoglobulin heavy variable 5-17 // 12|12 F2 // 780	Ighv5-17	OTTMUST0000013 0433	10
29259	17448807	---		---	10
27977	17434973	NM_013657 // Sema3c // sema domain, immunoglobulin domain (Ig), short basic domain, sec	Sema3c	NM_013657	10
12963	17287148	NM_001012324 // Ecm2 // extracellular matrix protein 2, female organ and adipocyte spec	Ecm2	NM_001012324	10
14063	17297125	NM_008981 // Ptprg // protein tyrosine phosphatase, receptor type, G // 14 6.33 cM|14 A	Ptprg	NM_008981	10
13680	17293165	NM_022317 // Slc28a3 // solute carrier family 28 (sodium-coupled nucleoside transporter	Slc28a3	NM_022317	10
34454	17497366	NM_001113414 // Ebf3 // early B cell factor 3 // 7|7 F3-F4 // 13593 /// NM_001113415 //	Ebf3	NM_001113414	10
12646	17284617	XR_865069 // Ighv1-62 // immunoglobulin heavy variable V1-62 // 12|12 F2 // 668542 ///	Ighv1-62	XR_865069	10
24303	17397750	NM_145539 // Tm4sf4 // transmembrane 4 superfamily member 4 // 3|3 D // 229302 /// ENSM	Tm4sf4	NM_145539	10
7816	17237028	NM_146240 // Rassf9 // Ras association (RalGDS/AF-6) domain family (N-terminal) member	Rassf9	NM_146240	10

17266	17328062	NM_007929 // Emp2 // epithelial membrane protein 2 // 16 A1|16 5.54 cM // 13731 /// ENS	Emp2	NM_007929	10
33884	17492314	NM_001045489 // Mfge8 // milk fat globule-EGF factor 8 protein // 7 D3|7 44.9 cM // 173	Mfge8	NM_001045489	10
21565	17369950	ENSMUST00000175536 // Gm26236 // predicted gene, 26236 [Source:MGI Symbol;Acc:MGI:54560	Gm26236	ENSMUST0000017 5536	10
19778	17352824	NM_145492 // Zfp521 // zinc finger protein 521 // 18 A1|18 7.68 cM // 225207 /// XM_006	Zfp521	NM_145492	10
36551	17517812	NM_011352 // Sema7a // sema domain, immunoglobulin domain (Ig), and GPI membrane anchor	Sema7a	NM_011352	10
12637	17284593	OTTMUST00000131094 // Ighv8-8 // immunoglobulin heavy variable 8-8 // 12|12 F2 // 78093	Ighv8-8	OTTMUST0000013 1094	10
32783	17481916	NM_020606 // Parva // parvin, alpha // 7|7 F1 // 57342 /// XM_006508050 // Parva // par	Parva	NM_020606	10
18129	17336681	NM_031176 // Tnxb // tenascin XB // 17 B1|17 18.24 cM // 81877 /// XM_006525185 // Tnxb	Tnxb	NM_031176	10
8999	17248426	NM_011412 // Slit3 // slit homolog 3 (Drosophila) // 11|11 A5 // 20564 /// ENSMUST00000	Slit3	NM_011412	10
19985	17354831	NM_001164491 // Ablim3 // actin binding LIM protein family, member 3 // 18|18 E1 // 319	Ablim3	NM_001164491	10
14628	17301452	NM_013492 // Clu // clusterin // 14 D1|14 34.36 cM // 12759 /// XM_006518503 // Clu //	Clu	NM_013492	10
11871	17278690	NR_030573 // Mir493 // microRNA 493 // 12|12 // 100124466	Mir493	NR_030573	10
21125	17365911	NM_001103177 // Ablim1 // actin-binding LIM protein 1 // 19 D2|19 52.09 cM // 226251 //	Ablim1	NM_001103177	10
15729	17312236	NM_026730 // Gpihbp1 // GPI-anchored HDL-binding protein 1 // 15|15 E1 // 68453 /// ENS	Gpihbp1	NM_026730	10
33302	17487422	NM_020486 // Bcam // basal cell adhesion molecule // 7|7 A3 // 57278 /// ENSMUST0000000	Bcam	NM_020486	10
30724	17463205	NM_011708 // Vwf // Von Willebrand factor homolog // 6 F3|6 59.32 cM // 22371 /// XM_00	Vwf	NM_011708	10
14649	17301634	XM_006519892 // Gm21451 // predicted gene, 21451 // 14|14 // 100862072 /// XM_006519893	Gm21451	XM_006519892	10
30201	17458362	NM_001243199 // Gimap4 // GTPase, IMAP family member 4 // 6|6 B2.3 // 107526 /// NM_001	Gimap4	NM_001243199	10
25534	17409792	NM_023245 // Palmd // palmdelphin // 3 G1|3 50.49 cM // 114301 /// ENSMUST00000040097 /	Palmd	NM_023245	10
30350	17459423	BC091754 // Igkv6-23 // immunoglobulin kappa variable 6-23 // 6|6 C1 // 637227 /// BC10	Igkv6-23	BC091754	10
6228	17220724	NM_008976 // Ptpn14 // protein tyrosine phosphatase, non-receptor type 14 // 1 H6|1 95.	Ptpn14	NM_008976	10
20530	17359689	NM_009128 // Scd2 // stearoyl-Coenzyme A desaturase 2 // 19 C3|19 37.98 cM // 20250 ///	Scd2	NM_009128	10
10604	17265164	NM_007528 // Bcl6b // B cell CLL/lymphoma 6, member B // 11|11 B4 // 12029 /// ENSMUST0	Bcl6b	NM_007528	10
37436	17527694	NM_010729 // Loxl1 // lysyl oxidase-like 1 // 9 B|9 31.65 cM // 16949 /// ENSMUST000000	Loxl1	NM_010729	10
21563	17369925	NM_001146348 // Eng // endoglin // 2 B|2 22.09 cM // 13805 /// NM_001146350 // Eng // e	Eng	NM_001146348	10
31208	17467389	OTTMUST00000132106 // Igkv10-94 // immunoglobulin kappa variable 10-94 // 6|6 C1 // 667	Igkv10-94	OTTMUST0000013 2106	10
22563	17379875	NR_028540 // Snord12 // small nucleolar RNA, C/D box 12 // 2|2 // 100217443 /// ENSMUST	Snord12	NR_028540	10
21840	17372644	NM_021398 // Slc43a3 // solute carrier family 43, member 3 // 2|2 E1 // 58207 /// XM_00	Slc43a3	NM_021398	10
14772	17302656	NM_001079844 // Gpc6 // glypican 6 // 14|14 E4 // 23888 /// NM_011821 // Gpc6 // glypic	Gpc6	NM_001079844	10
16294	17318089	NM_001252056 // Ly6c1 // lymphocyte antigen 6 complex, locus C1 // 15 D3|15 34.29 cM //	Ly6c1	NM_001252056	10
29111	17447835	NM_001163577 // Prom1 // prominin 1 // 5|5 B3 // 19126 /// NM_001163578 // Prom1 // pro	Prom1	NM_001163577	10
31244	17467489	OTTMUST00000132885 // Igkv5-43 // immunoglobulin kappa chain variable 5-43 // 6 C1|6 31	Igkv5-43	OTTMUST0000013 2885	10

SUPPLEMENTARY TABLE 1: Genes expressed at 10 times a higher level or greater in Glut2^{LO} cells vs Glut2^{HI} β-cells isolated from juvenile mouse pancreas
